# Knock-Out of Tenascin-C Ameliorates Ischemia-Induced Rod-Photoreceptor Degeneration and Retinal Dysfunction

**DOI:** 10.3389/fnins.2021.642176

**Published:** 2021-05-20

**Authors:** Susanne Wiemann, Aisha Yousf, Stephanie C. Joachim, Carolin Peters, Ana M. Mueller-Buehl, Natalie Wagner, Jacqueline Reinhard

**Affiliations:** ^1^Department of Cell Morphology and Molecular Neurobiology, Faculty of Biology and Biotechnology, Ruhr-University Bochum, Bochum, Germany; ^2^Experimental Eye Research Institute, University Eye Hospital, Ruhr-University Bochum, Bochum, Germany

**Keywords:** electroretinography, extracellular matrix, ischemia/reperfusion, retina, rod-bipolar cells, rod-photoreceptor cells, synapses, tenascin-C

## Abstract

Retinal ischemia is a common pathomechanism in various eye diseases. Recently, evidence accumulated suggesting that the extracellular matrix (ECM) glycoprotein tenascin-C (Tnc) plays a key role in ischemic degeneration. However, the possible functional role of Tnc in retinal ischemia is not yet known. The aim of our study was to explore retinal function and rod-bipolar/photoreceptor cell degeneration in wild type (WT) and *Tnc* knock-out (KO) mice after ischemia/reperfusion (I/R) injury. Therefore, I/R was induced by increasing intraocular pressure in the right eye of wild type (WT I/R) and *Tnc* KO (KO I/R) mice. The left eye served as untreated control (WT CO and KO CO). Scotopic electroretinogram (ERG) recordings were performed to examine rod-bipolar and rod-photoreceptor cell function. Changes of Tnc, rod-bipolar cells, photoreceptors, retinal structure and apoptotic and synaptic alterations were analyzed by immunohistochemistry, Hematoxylin and Eosin staining, Western blot, and quantitative real time PCR. We found increased Tnc protein levels 3 days after ischemia, while Tnc immunoreactivity decreased after 7 days. *Tnc* mRNA expression was comparable in the ischemic retina. ERG measurements after 7 days showed lower a-/b-wave amplitudes in both ischemic groups. Nevertheless, the amplitudes in the KO I/R group were higher than in the WT I/R group. We observed retinal thinning in WT I/R mice after 3 and 7 days. Although compared to the KO CO group, retinal thinning was not observed in the KO I/R group until 7 days. The number of PKCα^+^ rod-bipolar cells, recoverin^+^ photoreceptor staining and *Prkca* and *Rcvrn* expression were comparable in all groups. However, reduced rhodopsin protein as well as *Rho* and *Gnat1* mRNA expression levels of rod-photoreceptors were found in the WT I/R, but not in the KO I/R retina. Additionally, a lower number of activated caspase 3^+^ cells was observed in the KO I/R group. Finally, both ischemic groups displayed enhanced vesicular glutamate transporter 1 (vGlut1) levels. Collectively, KO mice showed diminished rod-photoreceptor degeneration and retinal dysfunction after I/R. Elevated vGlut1 levels after ischemia could be related to an impaired glutamatergic photoreceptor-bipolar cell signaling and excitotoxicity. Our study provides novel evidence that Tnc reinforces ischemic retinal degeneration, possibly by synaptic remodeling.

## Introduction

Retinal ischemia contributes to the pathophysiology of numerous eye disorders, such as vascular occlusions (e.g., central retinal vein/artery occlusion), acute glaucoma, and diabetic retinopathy (Osborne et al., 2004; Hayreh, 2005; Campochiaro, 2015). In the retina, ischemia develops as a result of capillary blockage and results in non-perfusion of this region. Just a few hours after ischemia, both inflammation and apoptosis occur. The mentioned diseases result in visual disturbances and possible blindness for these patients. A better understanding of the underlying pathomechanisms involved in retinal ischemia is therefore crucial for the development of novel diagnostic and therapy strategies to prevent vision loss.

Rodents represent useful *in vivo* systems to study the molecular mechanisms of ischemic neurodegeneration. Here, the ischemia/reperfusion (I/R) model represents a suitable experimental approach (Hartsock et al., 2016; Schultz et al., 2016; Joachim et al., 2017; Reinhard et al., 2017a; Renner et al., 2017; Palmhof et al., 2019). In the I/R model, retinal ischemia is induced by intraocular pressure (IOP) elevation for a definite period of time. Due to the induced high IOP, retinal blood vessels are compressed, which in turn leads to a loss or restriction of blood supply. The ischemic phase is followed by a natural reperfusion phase in which blood circulation is restored. This leads to further progressive damage of the retina through oxidative stress. Several studies indicate that ischemic neurodegeneration in the retina of different animal models is characterized by a loss of functional activity and loss of various neuronal subtypes (Kaur et al., 2008; Belforte et al., 2011; Joachim et al., 2012; Minhas et al., 2012; Schmid et al., 2014). I/R injury leads to the death of various retinal neurons, since they are sensitive to hypoxic stress (Kaur et al., 2008; Xu et al., 2015; Hu et al., 2016; Schultz et al., 2016; Palmhof et al., 2019).

There is increasing evidence that neurodegenerative processes are accompanied by an extensive remodeling of extracellular matrix (ECM) components (Roll et al., 2012; Roll and Faissner, 2014, 2019). The ECM forms a dynamic meshwork of macromolecules, termed matrisome, which is synthesized and secreted by the tissue-embedded cells themselves. Beside structural importance, the ECM regulates fundamental cellular processes such as adhesion, differentiation, migration, proliferation and survival. ECM components include fibrillary proteins, proteoglycans and glycoproteins. Especially, glycoproteins represent important receptor-, adhesion-, and adapter-molecules, which influence extra- as well as intracellular signaling pathways. Particularly, ECM molecules in the retina create a cellular environment that inhibits neuronal regeneration (Silver, 1994; Reinhard et al., 2015; Pearson et al., 2020). Nevertheless, the functional role of the ECM during ischemic processes and their impact on retinal neurodegeneration is not well known yet.

In the present study, we specifically focused on the functional importance of the ECM glycoprotein tenascin-C (Tnc) in retinal ischemia. During early retinogenesis, Tnc is first detectable at embryonic day 13 in post-mitotic cells of the inner neuroblastic layer (Klausmeyer et al., 2007). At this early stage, the knockout (KO) of *Tnc* led to a transient increase of post-mitotic neurons as well as an altered de-differentiation behavior of Müller glia *in vitro* (Besser et al., 2012). In the chicken retina, Tnc was reported to be expressed by horizontal, amacrine and displaced amacrine cells and in close association with the outer plexiform layer (OPL) and inner plexiform layer (IPL) (Bartsch et al., 1995; D’Alessandri et al., 1995). In the mouse retina, Tnc was also observed in colocalization with synaptophysin-immunoreactive synaptic sites of the plexiform layers (Reinhard et al., 2015). Additionally, optic nerve astrocytes can be a source of Tnc (Bartsch et al., 1992; Reinhard et al., 2015).

In the central nervous system (CNS), Tnc is a major functional constituent of the glial scar and has a harmful influence on neuronal cells after injury (Brodkey et al., 1995; Steindler et al., 1995; Kwok et al., 2011; Roll and Faissner, 2019). Interestingly, Tnc exhibits a low, if any expression in healthy tissue, while it is prominently upregulated following injury or in tumors (Faissner et al., 2017). Previously, evidence accumulated suggesting that Tnc is a key player in ischemic degeneration. In this regard, Tnc was previously described as crucial player in cerebral, hepatic as well as myocardial ischemia (Lu et al., 2003; Taki et al., 2010, 2015; Kuriyama et al., 2011).

Recently, we observed that Tnc and several interacting ECM molecules are dysregulated in a rat I/R model (Reinhard et al., 2017a, b). Tnc levels were reported to be increased in the optic nerve head of an ocular hypertension model (Johnson E. C. et al., 2007). Elevated Tnc expression could also be observed in the optic nerve head of patients with primary open-angle glaucoma (Pena et al., 1999). In a previous study, we also verified an upregulation of Tnc, and its interaction partner the chondroitin sulfate proteoglycan phosphacan in an experimental autoimmune and an IOP-dependent glaucoma mouse model (Reinehr et al., 2016; Reinhard et al., 2021). Immunized *Tnc* KO mice exhibit a reduced retinal ganglion cell loss and an enhanced anti-inflammatory cytokine expression (Wiemann et al., 2020), suggesting that *Tnc* deficiency provides various neuroprotective effects. However, the functional relevance of Tnc in retinal ischemia is poorly understood.

The goal of the present study was to comparatively explore retinal function, rod-bipolar/photoreceptor cell degeneration and synaptic alterations in WT and *Tnc* KO mice after I/R injury. Interestingly, we observed a diminished rod-photoreceptor degeneration and an improved retinal function in *Tnc* KO mice after ischemia. Additionally, we found increased vesicular glutamate transporter 1 (vGlut1) protein levels in both ischemic groups, which could be related to an impaired glutamatergic photoreceptor-bipolar cell signaling and excitotoxicity. Thus, the present study provides first evidence that Tnc accelerates ischemic rod-photoreceptor damage, maybe by modulating synaptic sites.

## Materials and Methods

### Mice

For all experiments, 6-week-old 129/Sv (129S2/SvPasCrl; background mouse strain) wild type (WT) and *Tnc* KO mice (Forsberg et al., 1996) of both genders were used. WT and *Tnc* KO mice were generated by the breeding of homozygous *Tnc* KO and WT mice, respectively. WT and *Tnc* KO breedings were derived from heterozygous breedings. Mouse colonies were maintained at the animal facility of the Faculty of Biology and Biotechnology, Ruhr-University Bochum (Bochum, Germany). Mice were housed under equal environmentally controlled lighting conditions (12 h light-dark cycle) with free access to chow and water.

### Genotyping

Genomic DNA was isolated from tail biopsies using the DirectPCR^®^ Lysis Reagent (Viagen Biotech Inc., Los Angeles, CA, United States). For genotyping, PCR analyses were performed using Taq DNA polymerase (Sigma-Aldrich St. Louis, MO, United States). The following primer pairs were used to amplify Tnc^+/+^ (WT) and Tnc^–/–^ (KO) alleles: Forward 5′-CTGCCAGGCATCTTTCTAGC-3′, reverse 5′-TTCTGCAGGTTGGAGGCAAC-3′ and neo forward 5′-CTGCTCTTTACTGAAGGCTC-3′ as described by Talts et al. (1999).

### Retinal Ischemia/Reperfusion

Before inducing retinal I/R, mice were anesthetized with a mixture of Medetomidine (500 μg/kg; Dorbene vet^®^ 1 mg/ml, Zoetis Deutschland GmbH, Berlin, Germany), Midazolam (5 mg/kg; Midazolam-hameln^®^ 1 mg/ml, hameln pharma plus GmbH, Hameln, Germany), and Fentanyl (50 μg/kg; Fentadon^®^ 50 μg/ml, Albrecht GmbH/Dechra Veterinary Products Deutschland GmbH, Aulendorf, Germany) by intraperitoneal injection. The cornea of the right eye of each animal was topically anesthetized with Oxybuprocaine hydrochloride (Novesine Stulln^®^ 4 mg/ml, Stulln pharma, Stulln, Germany) and the pupil was dilated with Tropicamide (Mydriaticum Stulln^®^ 5 mg/ml, Pharma Stulln, Stulln, Germany). A 27-gauge needle (Terumo Europe, Leuven, Belgium), which was connected by a flexible tube (B. Braun Intrafix SafeSet, Wolfram Droh GmbH, Mainz, Germany) to an NaCl reservoir (Isotonic saline solution 0.9% Ecoflac Plus, B. Braun Melsungen AG, Melsungen, Germany) was carefully inserted into the anterior chamber of the eye. By raising the saline reservoir, IOP was increased above a systolic blood pressure of 90 mmHg for 45 min. Ischemia was confirmed by blanching of the retina detected via an ophthalmoscope (Mini 3000, Heine Optotechnik, Herrsching, Germany). After removing of the needle, reperfusion of the retinal vasculature was observed by examining the fundus and the returning blood flow using an ophthalmoscope. Then, anesthesia was stopped by the subcutaneous application of Atipamezole (2.5 mg/kg; Alzane^®^ 5 mg/ml, Zoetis Deutschland GmbH, Berlin, Germany), Flumazenil (0.5 mg/kg; FLUMAzenil^®^ 0.1 mg/ml, B. Braun, Melsungen AG, Melsungen, Germany) and Naloxone (1200 μg/kg; Naloxon-hameln^®^ 0.4 mg/ml, hameln pharma plus GmbH, Hameln, Germany) cocktail. Non-ischemic left eyes served as controls. To avoid drying, control eyes were treated with a Dexpanthenol solution (Bepanthen^®^ Augen- und Nasensalbe 5 mg/g, Bayer Vital GmbH, Leverkusen, Germany). Ischemic eyes were treated with an Ofloxacin solution (Floxal^®^ 3 mg/ml, Bausch & Lomb GmbH, Heidelberg, Germany) and Corneregel^®^ (50 mg/g, Bausch & Lomb GmbH, Heidelberg, Germany). For pain therapy, metamizole (200 mg/kg) was administrated orally to drinking water for 4 days after ischemia. Eyes were inspected daily. Animals with signs of inflammation or cataract were excluded from the study. Animals were sacrificed by cervical dislocation at 3 and 7 days after I/R and retinae were explanted for immunohistochemistry, Western blot analyses and quantitative real time PCR (RT-qPCR).

### Electroretinogram Recordings

Scotopic full-field flash electroretinogram (ERG) recordings (HMsERG system, OcuScience, Henderson, NV, United States) were performed 7 days after I/R (*n* = 5/group) as described previously (Schmid et al., 2014; Reinhard et al., 2019). In brief, mice were dark-adapted overnight before performing ERG recordings under dim red light. Mice were anesthetized by an intraperitoneal injection of a ketamine/xylazine mixture (120/16 mg/kg). Eyes were treated with Oxybuprocaine hydrochloride eye drops (Novesine Stulln^®^ 4 mg/ml, Stulln pharma, Stulln, Germany) and the pupil was dilated with Tropicamide (Mydriaticum Stulln^®^ 5 mg/ml, Pharma Stulln, Stulln, Germany). Two reference electrodes were inserted subcutaneously behind the right and left ear and the ground electrode was placed in the base of the tail. Methocel (Omni Vision, Puchheim, Germany) was applied to the cornea and a contact lens with a silver-tread recording electrode (OcuScience, Henderson, NV, United States) was placed on the center of the cornea. Scotopic flash ERGs were recorded in non-ischemic control WT (WT CO) and KO (KO CO) as well as in ischemic WT (WT I/R) and KO (KO I/R) mice at 1.0, 3.0, 10.0, and 25.0 cd^∗^s/m^2^. From 1.0 to 3.0 cd^∗^s/m^2^, 4 flashes were averaged with an interstimulus interval of 10 s, while at 10.0 and 25.0 cd^∗^s/m^2^ 1 flash was measured. ERG responses were amplified, digitized, and analyzed using the ERGView 4.380R software (OcuScience) (Liu Y. et al., 2014). Before evaluating the a- and b-waves amplitudes, a 150 Hz low-pass filter was applied. In brief, a-waves were measured from the pre-stimulus baseline up to the a-wave, whereas b-waves were measured from the a-wave to the b-wave peak.

### Hematoxylin and Eosin Staining

At 3 and 7 days post-I/R, eyes were enucleated, fixed in 4% paraformaldehyde (PFA), dehydrated in 30% sucrose, and embedded in Tissue Freezing Medium^®^ (Leica, Buffalo Grove, IL, United States) for cross-sectioning (16 μm) with a cryostat (CM3050 S, Leica Mikrosysteme, Wetzlar, Germany). Hematoxylin and Eosin (H&E) staining of retinal sections was performed in accordance with the manufacturer’s protocol (Rapid Chrome H&E Frozen Section Staining Kit, Thermo Scientific). For thickness measurements, 2 central retinal sections per animal (*n* = 5/group) were analyzed. Brightfield images were captured with the Axio Zoom V16 (Carl Zeiss Microscopy, Jena, Germany) in central and mid-peripheral areas of each retinal section at a 200× magnification. Per image, measurements at 2 positions were performed using the Zeiss ZEN software (Carl Zeiss Microscopy). For total retinal thickness measurements, the distance from the basal ganglion cell layer (GCL) to the apical part of the outer nuclear layer (ONL) was measured. Additionally, we measured the thickness of the ONL and OPL alone and together (ONL + OPL). For layer measurements, the thickness of the WT CO group was set to 100%.

### Immunohistochemistry and Microscopy

For the preparation of retinal cross-sections, eyes were enucleated after 3 and 7 days and fixed in 4% PFA for 1 day (*n* = 5/group). After dehydration in sucrose (30%), eyes were embedded in Tissue Freezing Medium^®^ (Leica, Buffalo Grove, IL, United States). Retinal cross-sections (16 μm) were cut using a cryostat (CM3050 S, Leica Mikrosysteme, Wetzlar, Germany) and transferred onto Superfrost plus object slides (Menzel-Gläser, Braunschweig, Germany). Next, cross-sections were incubated in blocking solution [1% bovine serum albumin (BSA); Sigma-Aldrich; 3% goat serum, Dianova, Hamburg, Germany; 0.5% Triton^TM^-X-100, Sigma-Aldrich in 1 × phosphate-buffered saline (1 × PBS)] for 1 h at room temperature (RT). Then, the primary antibodies were diluted in blocking solution and incubated on sections overnight at RT (Table 1). Afterward, retinal cross-sections were washed in 1 × PBS and incubated for 2 h with species-specific secondary antibodies (Jackson Immuno Research Labs, Germany; Table 1) in blocking solution without Triton^TM^-X-100. Cell nuclei were counterstained using TO-PRO-3 (1:400; Thermo Fisher Scientific), which was diluted in secondary antibody solution.

**TABLE 1:** Primary and secondary antibodies used for immunohistochemistry.

Primary antibody	Dilution	Source/reference	RRID	Secondary antibody	Dilution	Source	RRID
Activated caspase 3	1:200	Sigma Aldrich	AB476884	Alexa Fluor 488-conjugated goat anti-rabbit	1:250	Jackson Immuno Research Labs	AB_2338049
Calbindin D-28k	1:500	Swant	AB_10000347	Alexa Fluor 488-conjugated goat anti-mouse	1:250	Jackson Immuno Research Labs	AB_2338844
GFAP	1:300	Sigma Aldrich	AB_477010	Alexa Fluor 488-conjugated goat anti-mouse	1:250	Jackson Immuno Research Labs	AB_2338844
PKCα	1:500	Santa Cruz	AB_628142	Alexa Fluor 488-conjugated goat anti-mouse	1:250	Jackson Immuno Research Labs	AB_2338844
Recoverin	1:600	Millipore	AB_2253622	Alexa Fluor 488-conjugated goat anti-rabbit	1:250	Jackson Immuno Research Labs	AB_2338049
Rhodopsin	1:600	Abcam	AB_304874	Cy3-conjugated goat anti-mouse	1:250	Jackson Immuno Research Labs	AB_2338686
Tnc (KAF12)	1:250	Wiemann et al., 2020	–	Cy3-conjugated goat anti-rabbit	1:250	Jackson Immuno Research Labs	AB_2338003

*Cy2 = Cyanine; Cy3 = Indocarbocyanine; RRID = Research Resource Identification number.*

Sections treated with secondary antibody only served as negative controls. Finally, sections were covered in Immu-Mount (Thermo Fisher Scientific) and stored at 4°C.

Immunostained retinal cross-sections were analyzed by confocal laser-scanning microscopy (LSM 510 META; Zeiss, Göttingen, Germany). For staining analyses, 2 central retinal sections per animal and 4 images per retina (2 mid-peripheral and two central) at a 400× magnification were captured. Laser lines and emission filters were adjusted using the Zeiss ZEN black software. Individual images were cropped with Coral Paint Shop Pro X8 (Coral Corporation, Gardena, CA, United States) and ImageJ software (ImageJ 1.51w, National Institutes of Health; Bethesda, MD, United States) was used to perform masked evaluation of the staining signal area (Supplementary Figure 1). Hence, images were converted into 32-bit gray scale. The background was subtracted, and lower and upper threshold values were determined for each image (Supplementary Table 1). Afterward, the percentage of the area fraction was measured by an ImageJ macro using antibodies directed against recoverin, rhodopsin and Tnc (Reinehr et al., 2018; Wiemann et al., 2020). Protein kinase alpha C (PKCα) cells were counted manually in a defined area of 225 × 225 μm using the cell counter tool of ImageJ. For these analyses, the staining area and the number of immunoreactive cells of the WT CO group was set to 100%. Activated caspase 3 immunoreactive cells were counted in the ONL of whole retinal sections.

### Western Blot

Western blot analyses were performed according to a previously described protocol (Reinhard et al., 2017a). Ischemic and control retinae of both genotypes (*n* = 4/group) were homogenized in 80 μl lysis buffer containing 60 mM n-octyl-β-D-glucopyranoside, 50 mM sodium acetate, 50 mM Tris chloride, pH 8.0 and a protease inhibitor cocktail (Sigma-Aldrich) for 1 h on ice. After centrifugation at 14.000 × *g* at 4°C for 30 min, protein concentration in the supernatant was determined with the bicinchoninic acid assay (BCA) Protein Assay Kit (Pierce, Thermo Fisher Scientific) according to manufacturer’s instructions. 4 × SDS buffer was added to each protein sample (15–20 μg) and denaturized for 5 min at 94°C. Following separation by SDS-PAGE using 4–12% polyacrylamide gels, proteins were transferred to a polyvinylidene difluoride (PVDF) membrane (Roth, Karlsruhe, Germany) via Western blotting. Next, membranes were blocked in TBST (5% w/v milk powder in Tris-buffered saline (TBS) and 0.05% Tween 20) at RT for 1 h. Primary antibodies (Table 2) were diluted in blocking solution and membranes were incubated in the solution at 4°C overnight. Membranes were washed in TBST and incubated with horseradish peroxidase (HRP) conjugated secondary antibodies (Table 2) in blocking solution at RT for 2 h. Non-bound antibody was washed off with TBST and finally in TBS. ECL substrate solutions were mixed 1:1 (Bio-Rad Laboratories GmbH, München, Germany) and applied to the membranes for 5 min. Immunoreactivity of proteins was recorded with a MicroChemi Chemiluminescence Reader (Biostep, Burkhardtsdorf, Germany). Protein band intensity was measured using ImageJ software and normalized to the internal reference proteins actin and α-tubulin (Table 2). The normalized values of the Western blot results were presented in arbitrary units (a.u.). Tnc protein was immunoaffinity-purified from postnatal mouse brains using the monoclonal Tnc antibodies J1/tn1 (clone number 576, rat, IgG), J1/tn2 (clone number 578, rat, IgG_2__*a*_), and J1/tn4 (clone number 633, rat, IgG_2__*a*_) as previously described (Faissner and Kruse, 1990; Husmann et al., 1992) (Supplementary Figure 2).

**TABLE 2:** Primary and secondary antibodies used for Western blot analyses.

Primary antibody	Dilution	Source/reference	RRID	Molecular weight (kDa)	Secondary antibody	Dilution	Source	RRID
α-Tubulin	1:10.000	Sigma Aldrich	AB_477593	∼50	Peroxidase-conjugated goat anti-mouse	1:10.000	Jackson Immuno Research Labs	AB_2338505
Actin	1:5.000	BD Biosciences	AB_399901	42	Peroxidase-conjugated goat anti-mouse	1:10.000	Jackson Immuno Research Labs	AB_2338505
CtBP2	1:2.000	BD Biosciences	AB_399431	48	Peroxidase-conjugated goat anti-mouse	1:10.000	Jackson Immuno Research Labs	AB_2338505
PKCα	1:500	Santa Cruz	AB_628142	80	Peroxidase-conjugated goat anti-mouse	1:10.000	Jackson Immuno Research Labs	AB_2338505
Recoverin	1:1.000	Millipore	AB_2253622	23	Peroxidase-conjugated goat anti-rabbit	1:10.000	Jackson Immuno Research Labs	AB_2307391
Rhodopsin	1:10.000	Abcam	AB_304874	40	Peroxidase-conjugated goat anti-mouse	1:10.000	Jackson Immuno Research Labs	AB_2338505
Tnc (KAF12)	1:5.000–1:10.000	Wiemann et al., 2020	–	∼250 and >250 kDa	Peroxidase-conjugated goat anti-rabbit	1:10.000	Jackson Immuno Research Labs	AB_2307391
vGlut1	1:2000	Sigma Aldrich	AB_261840	60	Peroxidase-conjugated goat anti-rabbit	1:10.000	Jackson Immuno Research Labs	AB_2307391

*kDa = kilo Dalton; RRID = Research Resource Identification number.*

### RNA Isolation, cDNA Synthesis and RT-qPCR Analysis

Retinae were explanted at 3 and 7 days after I/R and stored at −80°C (*n* = 4–5/group). RNA was extracted according to the manufacturer’s instructions using the Gene Elute Mammalian Total RNA Miniprep Kit (Sigma-Aldrich, St. Louise, MO, United States). RNA quality and quantity were analyzed using a BioSpectrometer^®^ (Eppendorf, Hamburg, Germany). Via reverse transcription with random hexamer primers, 1 μg RNA was used for cDNA synthesis (Thermo Fisher Scientific). Forward and reverse primer was designed using the Universal ProbeLibrary Assay Design Center software (Roche Molecular Systems; Table 3). For each primer set efficiencies were determined by a dilution series of 5, 25, and 125 ng cDNA. RT-qPCR analyses were performed with SYBR Green I in a Light Cycler 96^®^ (Roche Applied Science, Mannheim, Germany) as described previously (Reinhard et al., 2019). Expression was normalized against the constantly expressed housekeeping gene *hypoxanthine guanine phosphoribosyl transferase* (*Hprt*).

**TABLE 3:** List of primer pairs used for RT-qPCR analyses.

Primer	Sequence	Product size (base pairs)	Primer efficiency	GenBank accession number
*Cacna1f*_forward	TGTGTGCAGATGGTCCTTATATCT	78	0.685	NM_019582
*Cacna1f*_reverse	CTGGGCTCTGGGGTTGTAT			
*Gnat1*_forward	GAGGATGCTGAGAAGGATGC	106	0.806	NM_008140
*Gnat1*_reverse	CGTCCTGGTGGATAATCTTCA			
*Hprt*_forward	TGATAGATCCATTCCTATGACTGTAGA	126	0.931	NM_013556 XM_356404
*Hprt*_reverse	AAGACATTCTTTCCAGTTAAAGTTGAG			
*Prkca*_forward	CAAGGGATGAAATGTGACACC	96	0.980	NM_011101.3
*Prkca*_reverse	CCTCTTCTCTGTGTGATCCATTC			
*Rcvrn*_forward	CAATGGGACCATCAGCAAA	71	0.836	NM_009038.2
*Rcvrn*_reverse	CCTCAGGCTTGATCATTTTGA			
*Rho*_forward	ACCTGGATCATGGCGTTG	70	0.934	NM_145383.1
*Rho_reverse*	TGCCCTCAGGGATGTACC			
*Tnc_forward*	CAGGGATAGACTGCTCTGAGG	90	1.0	NM_001369211–14
*Tnc_reverse*	CATTGTCCCATGCCAGATTT			

### Statistical Analysis

Pairwise analyses of Tnc protein levels in two groups (WT CO vs. WT I/R) were carried out with the Student *t*-test (Statistica software, V13.3; StatSoft Europe, Hamburg, Germany).

For a multiple comparison of all four groups (WT CO, WT I/R, KO CO, and KO I/R), ERG data, histological stainings and Western blots were analyzed using a two-way ANOVA followed by Tukey’s *post hoc* test.

A pairwise fixed reallocation and randomization test (REST software, Relative expression software tool 2009; QIAGEN GmbH, Hilden, Germany) was used to analyze RT-qPCR data.

## Results

### Early Induction of Tnc Following Retinal Ischemia

**FIGURE 1:**
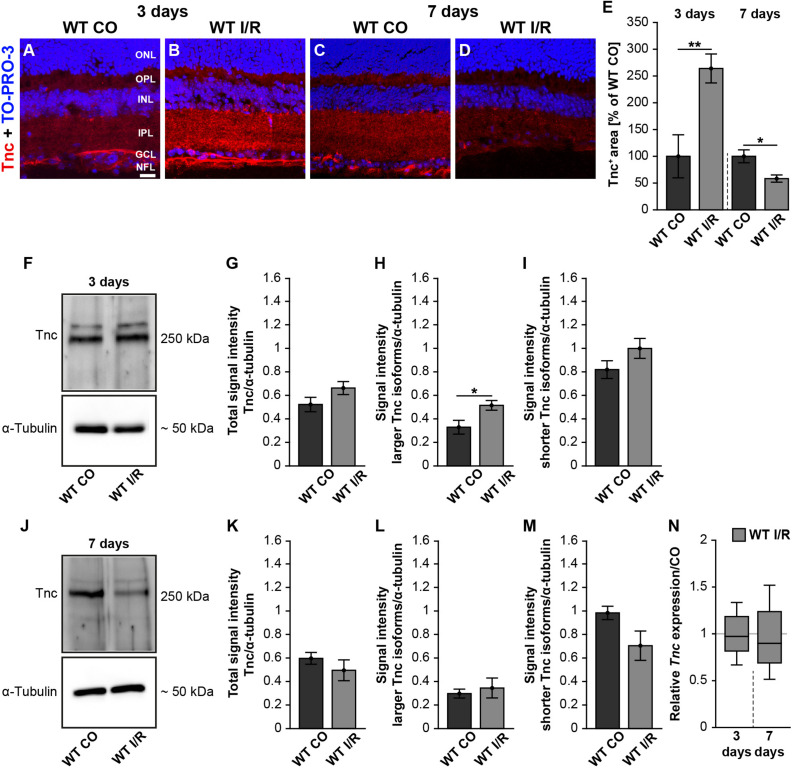
Early induction of Tnc in the ischemic WT retina. **(A–D)** Images of Tnc stained retinal cross-sections from control and ischemic WT groups at 3 and 7 days. Immunohistochemistry revealed a Tnc signal in the IPL and OPL as well as in the NFL (red). Cell nuclei were counterstained with TO-PRO-3 (blue). **(E)** Tnc^+^ staining area was significantly increased at 3 days, indicating an early induction of Tnc after ischemic injury. However, a significantly reduced Tnc staining could be demonstrated at 7 days. The WT CO group was set to 100%. **(F–I)** Consistently, relative protein quantification showed a significant upregulation of larger Tnc isoforms at 3 days after I/R. **(J–M)** Comparable Tnc protein levels were observed after 7 days. **(N)** No differences of the *Tnc* mRNA expression were noted at both points in time after ischemia. Data were analyzed via Student *t*-test and presented as mean ± standard error mean (SEM) in panels **(E,G–I,K–M)**. For RT-qPCR, groups were compared using the pairwise fixed reallocation and randomization test in panel **(N)**. These data are shown as median ± quartile ± minimum/maximum. *p < 0.05; **p < 0.01. *n* = 4–5/group. Scale bar = 20 μm. ONL: outer nuclear layer, OPL: outer plexiform layer, INL: inner nuclear layer, IPL: inner plexiform layer, GCL: ganglion cell layer, NFL: nerve fiber layer.

It is not known whether Tnc is dysregulated in the retina of our I/R mouse model. Therefore, we analyzed the Tnc expression pattern in non-ischemic and ischemic WT retinae after 3 and 7 days (Figure 1). Immunohistochemistry of retinal cross-sections revealed an obvious Tnc immunoreactivity in the OPL and IPL as well as in close association with cells of the inner nuclear layer (INL) and nerve fiber layer (NFL) (Figures 1A–D). At 3 days after ischemia, we found a significant increase of the Tnc^+^ staining area (*p* < 0.01; Figure 1E). Conversely, a decreased Tnc^+^ staining area was noted in ischemic WT retinae at 7 days (*p* = 0.02; Figure 1E).

In order to analyze the expression pattern of Tnc after ischemia, we performed coimmunostaining (Supplementary Figure 3). Here, anti-calbindin was used as a specific marker for horizontal and amacrine cells, while anti-glial fibrillary acidic protein (GFAP) was used to label astrocytes. Calbindin- and Tnc-double-positive horizontal cells were observed in the most apical part of the INL. Tnc immunoreactivity in the basal part of the INL was also found in close association with calbindin^+^ amacrine cells. In addition, we revealed a colocalization of GFAP^+^ astrocytes and Tnc in the NFL, indicating that the mentioned cells are a source of Tnc expression in the retina.

To consolidate the dysregulation of Tnc after ischemia, we verified Tnc protein levels by Western blot analyses. For Tnc, two distinct bands were observed at ∼250 and >250 kDa (Figures 1F,J and Supplementary Figure 2). At 3 days, densiometric measurements of the total Tnc protein showed comparable levels in WT CO and WT I/R retinae (*p* = 0.13; Figure 1G). Interestingly, a significant upregulation of the larger Tnc isoforms was found in the WT I/R compared to the WT CO group (*p* = 0.03; Figure 1H). Unaltered protein levels were observed for shorter Tnc isoforms in both groups (*p* = 0.15; Figure 1I). At 7 days, total Tnc protein levels were unaltered (*p* = 0.36; Figure 1K). Also, protein levels of larger Tnc isoforms were comparable in control and ischemic WT retinae (*p* = 0.63; Figure 1L). Relative quantification showed a slight, but not statistically significant, decrease of shorter Tnc isoforms after I/R compared to the control condition (*p* = 0.09; Figure 1M).

Finally, we examined *Tnc* mRNA levels using RT-qPCR at both points in time. However, compared to the non-ischemic condition, *Tnc* mRNA expression was similar at 3 (*p* = 0.78) and 7 days (*p* = 0.58) after ischemia (Figure 1N and Supplementary Table 2).

Collectively, our temporal analyses of Tnc induction revealed a very early raise followed by a decrease of the Tnc protein level after ischemic damage, while *Tnc* mRNA expression was unaltered. Accordingly, our results indicate that the upregulation of Tnc is a very early event during retinal ischemic damage.

### Improvement of Retinal Functionality in *Tnc* KO Mice After I/R

**FIGURE 2:**
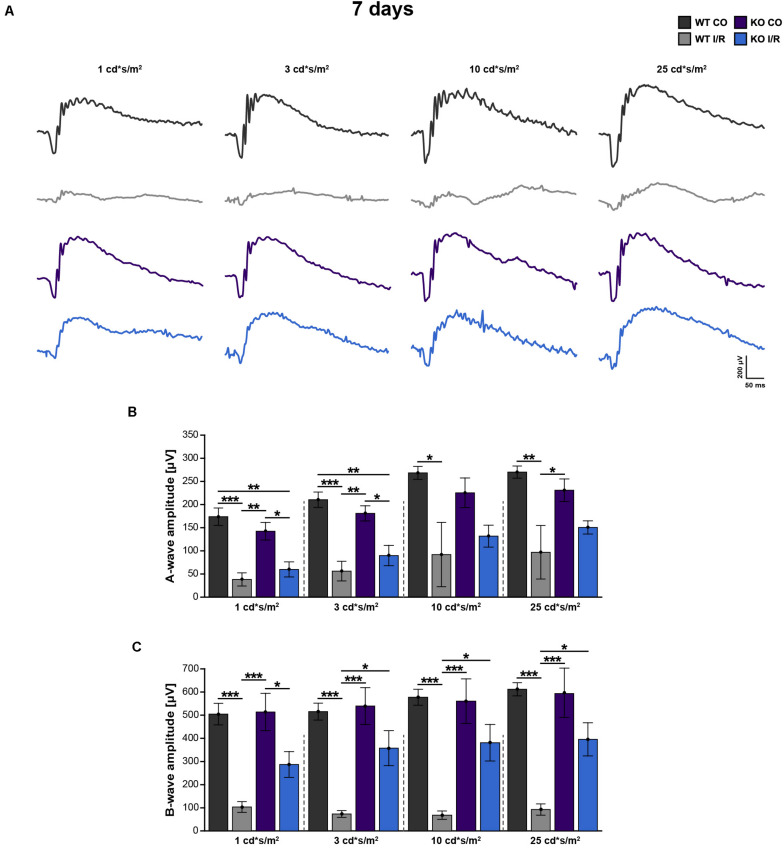
Improvement of retinal function in ischemic *Tnc* KO mice. **(A–C)** Retinal function was analyzed in the control WT (WT CO) and *Tnc* KO (KO CO) groups as well as in the ischemic WT (WT I/R) and *Tnc* KO groups (KO I/R) at 7 days after I/R. **(A)** Exemplary ERG waveforms for all groups at the flash luminances of 1, 3, 10, and 25 cd*s/m^2^ are shown. **(B,C)** The flash luminance (cd*s/m^2^) and the a- or b-wave amplitude size (μV) are indicated in the graphs. Significant lower a- and b-wave amplitudes were found in both genotypes after I/R. However, the KO I/R group showed significantly reduced a-wave amplitudes only at lower flash luminances in comparison to the control KO group. Interestingly, in the KO I/R group, b-wave amplitudes from 3 to 25 cd*s/m^2^ were significantly higher in comparison to the WT I/R group. *p < 0.05, **p < 0.01, ***p < 0.001. *n* = 5/group. Groups were analyzed using two-way ANOVA followed by Tukey’s *post hoc* test. Data are shown as mean ± SEM. cd: candela; μV: micro volt; m: minutes; s: seconds.

To examine possible functional deficits in control and ischemic WT and *Tnc* KO retinae, scotopic ERG-recordings were conducted at 7 days post-I/R (Figures 2A–C and Supplementary Table 3). In all experimental groups, increased a- and b-wave amplitudes were observed with rising light flash stimuli. Rod-photoreceptor cell and rod-bipolar cell responses were evaluated by a- and b-wave amplitude measurements, respectively.

Compared to the WT CO group, significantly reduced a-wave (*p* < 0.05) and b-wave (*p* < 0.01) amplitudes were recorded in the WT I/R condition. Also, the KO I/R group showed significantly reduced a-wave responses at lower light stimuli in comparison to the KO CO group (*p* < 0.05 from 1 to 3 cd^∗^s/m^2^). However, although the amplitudes of the a-wave were comparable in both ischemic groups (*p* > 0.05), higher amplitudes were observed in the KO I/R group.

The KO I/R and KO CO groups showed similar b-wave amplitudes (*p* ≥ 0.05). Compared to the KO I/R group, the WT I/R group exhibited significantly reduced b-wave responses (*p* < 0.05 from 3 to 25 cd^∗^s/m^2^).

In summary, although the KO I/R group was less affected, impaired a-wave responses were observed in both genotypes after ischemia. Even though, in relation to the WT group, the KO group showed an impaired, but much better b-wave functionality following ischemia. Collectively, our results suggest that the loss of Tnc acts neuroprotective after I/R on a functional level.

### Early Preservation of Retinal Integrity in the KO I/R Retina

**FIGURE 3:**
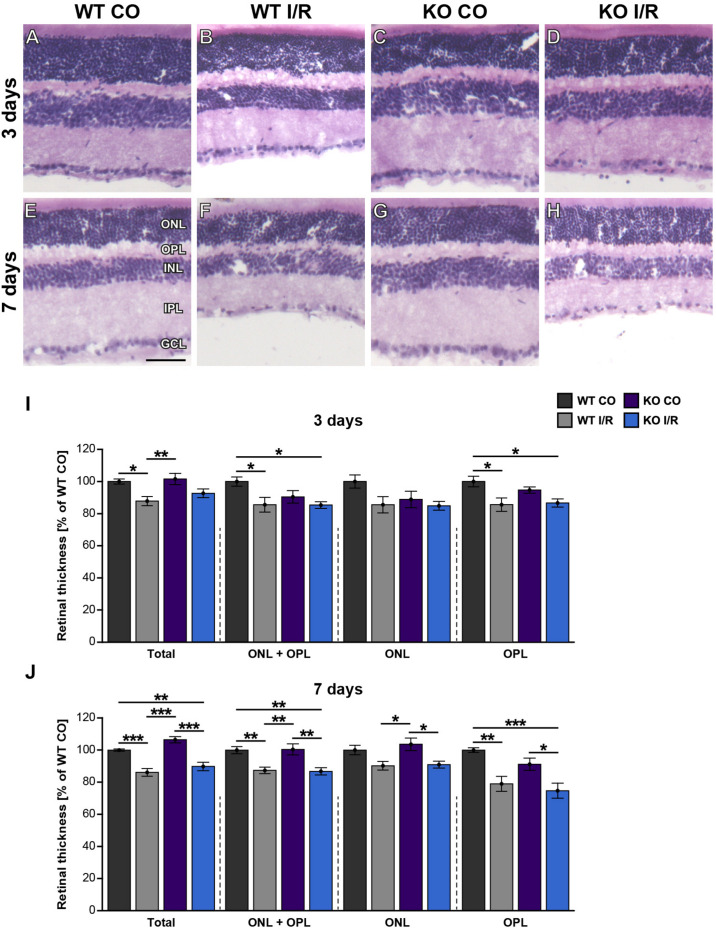
Maintenance of early retinal integrity in *Tnc* KO mice. **(A–H)** Cross-sections of control and ischemic WT and KO retinae were stained with H&E at 3 and 7 days post-I/R. **(I)** At 3 days, a significant reduction of the total, ONL and OPL as well as OPL retinal thickness was noted in the WT I/R group compared to the WT CO group. However, overall outer retinal thickness was comparable in the KO I/R and KO CO group, suggesting an improved structural retinal integrity in the KO early after ischemia. **(J)** At 7 days, a comparable, progressive decline in the outer retinal thickness was observed in both ischemic groups. Groups were analyzed using two-way ANOVA followed by Tukey’s *post hoc* test. Data are displayed as mean ± SEM in panels **(I,J)**. The WT CO group was set to 100%. *p < 0.05, **p < 0.01, ***p < 0.01. *n* = 5/group. Scale bar = 50 μm. ONL: outer nuclear layer, OPL: outer plexiform layer, INL: inner nuclear layer, IPL: inner plexiform layer, GCL: ganglion cell layer.

In a further step, the structural integrity of the retina should be analyzed in WT and KO mice after ischemia. Thus, retinal cross-sections of all groups were stained with H&E (Figures 3A–H). Then, the thickness of the total retina, the ONL and OPL together as well as the ONL and the OPL separately were measured. At 3 days, the non-ischemic control retinae were well defined and overall retinal thickness was comparable in both genotypes (WT CO vs. KO CO, *p* = 0.23; Figure 3I). Conversely, compared to the WT CO group, total retinal thickness, ONL + OPL thickness as well as OPL thickness were significantly reduced in the WT I/R group (WT CO vs. WT I/R, total: *p* = 0.02; ONL + OPL: *p* = 0.03; OPL: *p* = 0.01), while no differences were found in the ONL (*p* > 0.11). Interestingly, no significant reduction in the layer thickness was observed in the KO I/R group compared to the KO CO group (KO CO vs. KO I/R, *p* = 0.13). Although ONL + OPL (*p* = 0.03) as well as OPL thickness (*p* = 0.02) was reduced in the KO I/R compared to the WT CO state.

At 7 days, the thickness of retinae from WT CO and KO CO animals was quite similar (WT CO vs. KO CO, *p* > 0.05; Figure 3J). Overall, retinal thickness of both ischemic groups was reduced compared to the control groups (WT CO vs. WT I/R, total: *p* < 0.001; ONL + OPL: *p* = 0.008; ONL: *p* = 0.13; OPL: *p* = 0.006 and KO CO vs. KO I/R, total: *p* < 0.001; ONL + OPL: *p* = 0.004; ONL: *p* = 0.03; OPL: *p* = 0.03). In comparison to the WT CO group, the KO I/R group showed a significant total (*p* = 0.008), ONL and OPL (*p* = 0.005) and OPL (*p* < 0.001) layer reduction. Also, total (*p* < 0.001), ONL + OPL (*p* = 0.006) as well as the OPL (*p* = 0.02) was significantly thinner in the WT I/R compared to the KO CO condition. Similar retinal layer thicknesses were measured in both ischemic conditions (*p* = 0.14).

Collectively, we observed a reduced retinal thickness in the WT group at 3 and 7 days after ischemia. Strikingly, retinal thickness was not affected in the KO group at 3 days post-I/R. *Tnc* deficient mice showed a reduced retinal thickness only at 7 days after ischemia. These data point to a delayed retinal degeneration and early maintenance of retinal integrity in the *Tnc* KO. In addition, our data indicate that the structural integrity of the OPL is in particular severely impaired after retinal ischemia, while the integrity of the ONL is less affected at both examined time points.

### Bipolar Cells Are Not Affected by Ischemia in WT and *Tnc* KO Mice

**FIGURE 4:**
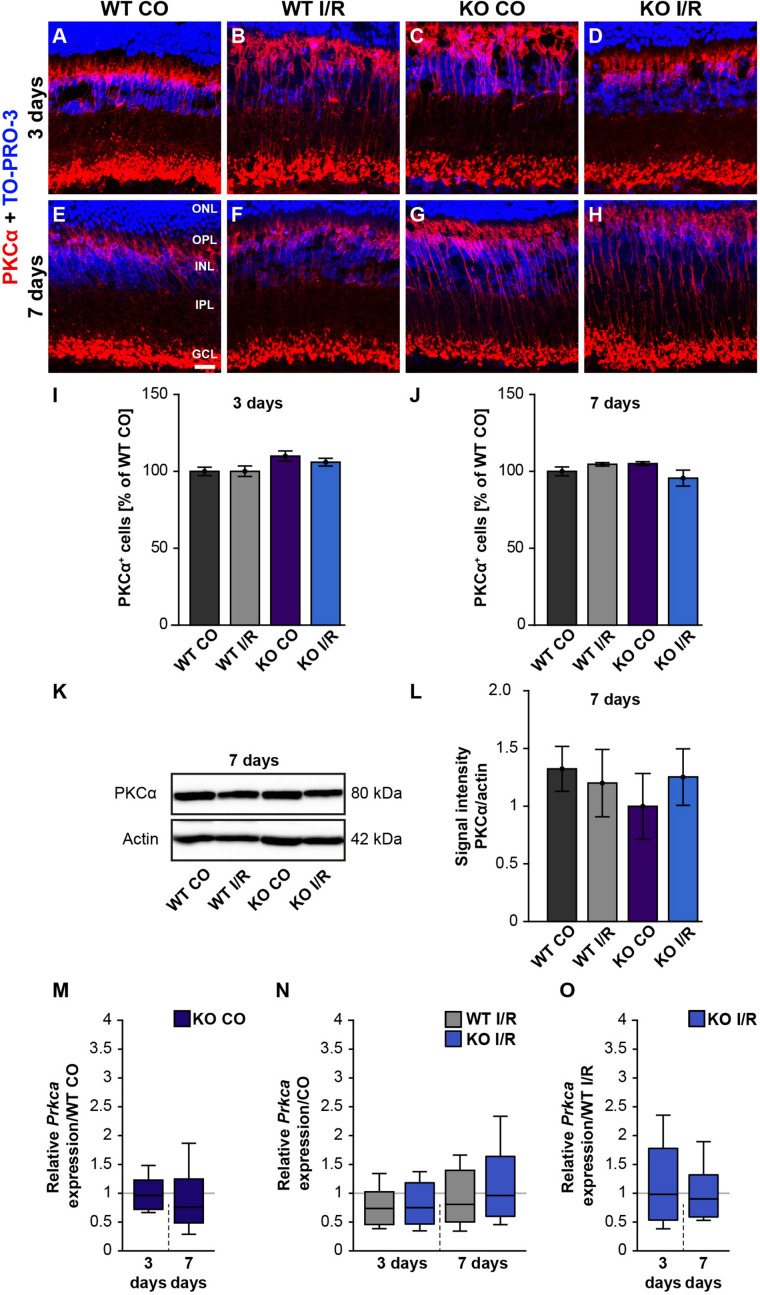
Rod-bipolar cells are not affected early ischemia. **(A–H)** Anti-PKCα was used to label rod-bipolar cells (red) 3 and 7 days post-I/R. TO-PRO-3 (blue) was used as nuclear counterstain. **(I,J)** Countings of PKCα^+^ cells demonstrated no changes between control and ischemic WT and KO retinae 3 and 7 days post-I/R. The WT CO group was set to 100%. **(K,L)** In line with the immunohistochemical results, comparable protein levels of PKCα were detected via Western blot in all four groups 7 days post-I/R. **(M–O)** RT-qPCR analyses showed an unaltered *Prkca* mRNA expression level in all groups at both points in time after ischemia. Groups were evaluated using two-way ANOVA followed by Tukey’s *post hoc* test. Data are indicated as mean ± SEM in panels **(I,J,L)**. For RT-qPCR results, groups were analyzed using the pairwise fixed reallocation and randomization test. Data are shown as median ± quartile ± minimum/maximum in panels **(M–O)**. *p* > 0.05. *n* = 4–5/group. Scale bar = 20 μm. ONL: outer nuclear layer; OPL: outer plexiform layer; INL: inner nuclear layer; IPL: inner plexiform layer, GCL: ganglion cell layer.

In order to analyze whether rod-bipolar cells are affected in both genotypes after ischemic injury, we performed PKCα immunostainings. However, the number of PKCα^+^ cells in control and ischemic retinae of both genotypes was similar at 3 days (WT CO vs. WT I/R, *p* = 0.99; KO CO vs. KO I/R, *p* = 0.79; Figures 4A–D,I) as well as at 7 days post-I/R (WT CO vs. WT I/R, *p* = 0.72; KO CO vs. KO I/R, *p* = 0.18; Figures 4E–H,J).

In line with these results, total PKCα protein levels, investigated by Western blotting at 7 days, were equivalent in all groups (WT CO vs. WT I/R, *p* = 0.99; KO CO vs. KO I/R, *p* = 0.90; Figures 4K,L).

RT-qPCR results showed no differences in the *Prkca* mRNA-expression between both control groups (WT CO vs. KO CO, *p* = 0.78; Figure 4M and Supplementary Table 2) at 3 days post-I/R. *Prkca* mRNA level in WT CO and KO CO animals resembled the expression level in the WT I/R (WT CO vs. WT I/R, *p* = 0.18) and the KO I/R group (KO CO vs. KO I/R, *p* = 0.20; Figure 4N). A similar *Prkca* expression was found in both ischemic groups (WT I/R vs. KO I/R, *p* = 0.91; Figure 4O). Also, at 7 days, RT-qPCR results verified no changes in the *Prkca* mRNA expression between the control and the ischemic groups (WT CO vs. KO CO, *p* = 0.32, Figure 4M; WT CO vs. WT I/R, *p* = 0.35, KO CO vs. KO I/R, *p* = 0.86; Figure 4N and WT I/R vs. KO I/R, *p* = 0.60; Figure 4O).

In conclusion, no differences regarding the rod-bipolar cell integrity were observed in WT and *Tnc* KO retinae after I/R. Therefore, we verified a preservation of the PKCα^+^ rod-bipolar cell population in both genotypes following ischemic injury.

### Less Vulnerability of Rod-Photoreceptors in Ischemic KO Mice

**FIGURE 5:**
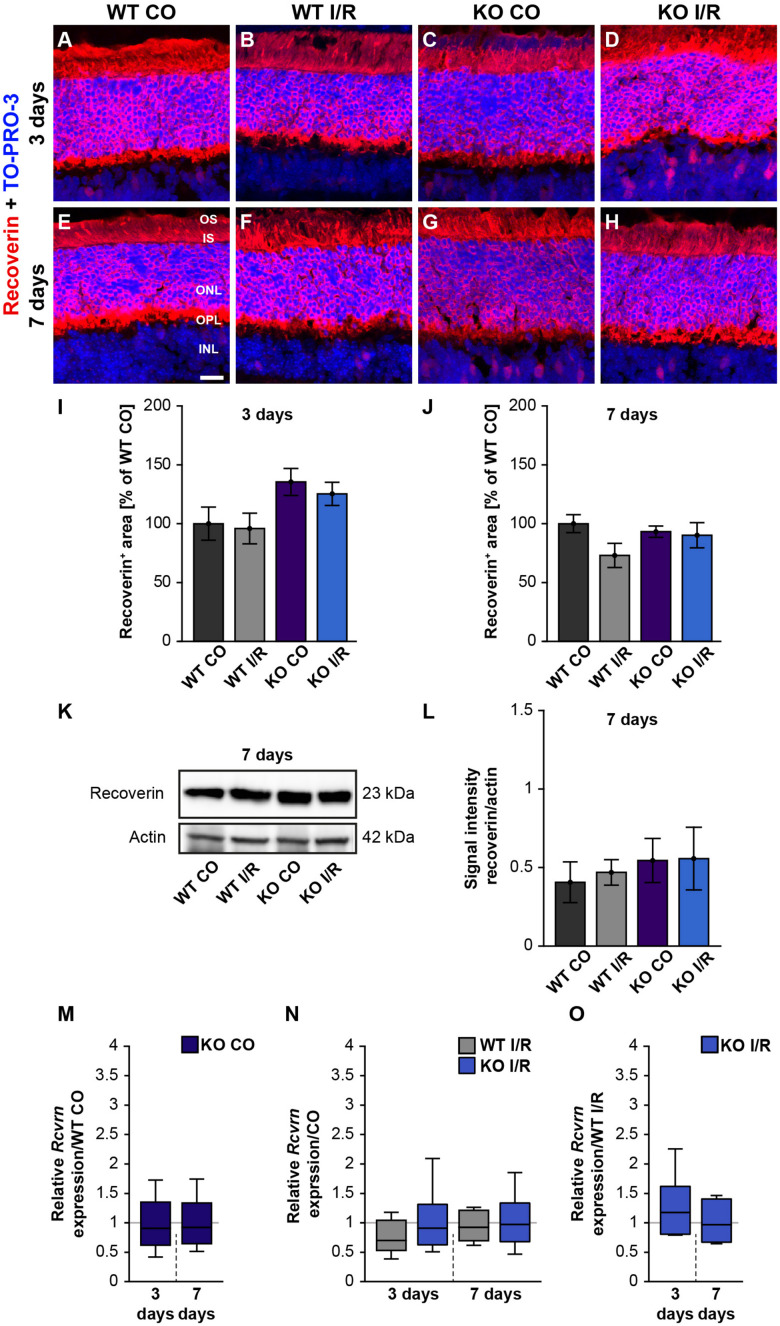
Unaltered recoverin levels in WT and KO mice after ischemia. **(A–H)** Photoreceptors cells were marked with an antibody against recoverin (red) and cell nuclei were labeled with TO-PRO-3 (blue) at 3 and 7 days post-I/R. **(I,J)** Recoverin^+^ staining area was similar in the control and ischemic WT and KO groups at both analyzed points in time. The WT CO group was set to 100%. **(K)** Exemplary Western blots of recoverin at 7 days post-I/R. **(L)** No changes in the recoverin band intensity were observed in both genotypes. **(M)** A comparable *Rcvrn* expression was seen in the non-ischemic WT and KO groups at 3 as well as 7 days post-I/R. **(N)** An equal mRNA level of *Rcvrn* was detected in the ischemic groups when compared to the corresponding control groups. **(O)** In comparison to the WT I/R group, we also observed an unaltered *Rcvrn* expression in the KO I/R group. Groups were analyzed using two-way ANOVA followed by Tukey’s *post hoc* test. Data are shown as mean ± SEM in panels **(I,J,L)**. For RT-qPCR analyses, groups were compared using the pairwise fixed reallocation and randomization test. Data are shown as median ± quartile ± minimum/maximum in panels **(M–O)**. *p* > 0.05. *n* = 4–5/group. Scale bar = 20 μm. OS: outer segments; IS: inner segments; ONL: outer nuclear layer; OPL: outer plexiform layer; INL: inner nuclear layer.

Next, we analyzed photoreceptor cells by recoverin staining, which was localized in the ONL as well as in the inner and outer photoreceptor segments (Figures 5A–H). The recoverin^+^ staining area was comparable in both WT groups (WT CO vs. WT I/R, *p* = 0.99) as well as in the KO condition (KO CO vs. KO I/R, *p* = 0.94; Figure 5I) at 3 days after ischemia. An unaltered recoverin^+^ staining area was also found in non-ischemic and ischemic WT and KO retinae at 7 days (WT CO vs. WT I/R, *p* = 0.17 and KO CO vs. KO I/R, *p* = 0.99; Figure 5J).

Furthermore, recoverin (23 kDa) was quantified via Western blot analyses (Figure 5K). The statistical evaluation revealed equal protein levels in WT as well as KO mice (WT CO vs. WT I/R, *p* = 0.99 and KO CO vs. KO I/R, *p* = 1.0; Figure 5L).

We also determined the expression levels of *recoverin* (*Rcvrn*) in control and ischemic WT and KO retinae at both points in time (Figures 5M–O and Supplementary Table 2). Non-ischemic eyes exhibited equal *Rcvrn* mRNA levels at both points in time (3 days: WT CO vs. KO CO, *p* = 0.64; 7 days: WT CO vs. KO CO, *p* = 0.67; Figure 5M). The RT-qPCR results also offered comparable expression levels between the non-ischemic control and ischemic WT groups (3 days: WT CO vs. WT I/R, *p* = 0.07; 7 days: WT CO vs. WT I/R, *p* = 0.52) as well as KO groups (3 days: KO CO vs. KO I/R, *p* = 0.62; 7 days: KO CO vs. KO I/R, *p* = 0.84; Figure 5N). Additionally, we observed a similar *Rcvrn* expression between the genotypes 3 days (WT I/R vs. KO I/R; *p* = 0.32) and at 7 days post-ischemia (WT I/R vs. KO I/R, *p* = 0.57; Figure 5O).

**FIGURE 6:**
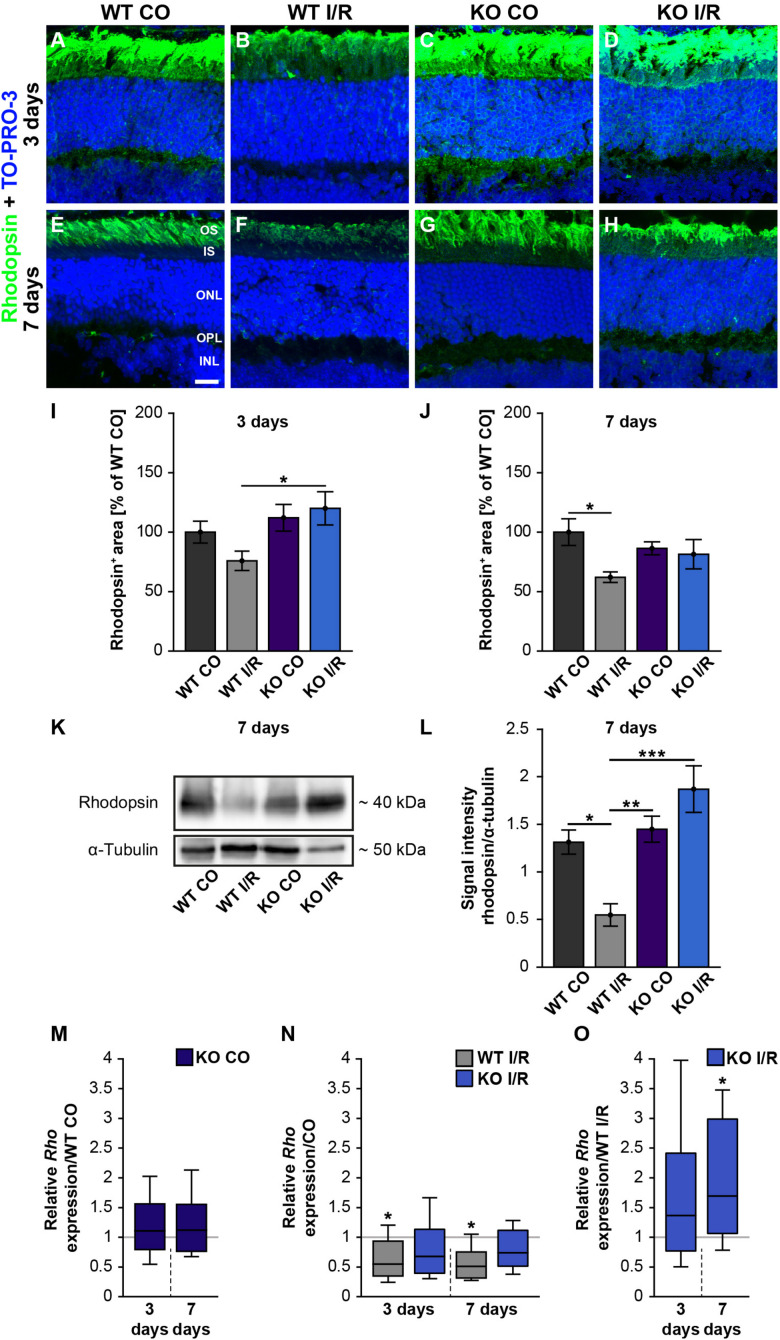
Preservation of rod-photoreceptor cells in the ischemic *Tnc* KO retina. **(A–H)** Rod-photoreceptors were labeled with rhodopsin (green) in control and ischemic WT and KO retinae 3 and 7 days after I/R. TO-PRO-3 (blue) was used as nuclear counterstain. **(I)** At 3 days, a significantly decreased rhodopsin staining area was only noted in the ischemic WT group, but not in the ischemic KO group. Moreover, the rhodopsin staining was significantly increased in the KO I/R group when compared to the WT I/R group. **(J)** At 7 days post-I/R, we detected a significant reduction of the rhodopsin-stained area in the WT I/R compared to the WT CO group. However, the KO I/R group showed a comparable rhodopsin staining as the KO CO group. **(K)** Analyses of rhodopsin via Western blot. **(L)** Quantification of band intensity revealed reduced protein levels in the WT I/R group when compared to the other groups at 7 days post-I/R. **(M)** No difference in the *Rho* expression was noted in the KO CO group compared to the WT CO group at both points in time. **(N)** A significant downregulation of the *Rho* mRNA level was seen in the WT I/R group in comparison to the WT CO group at 3 and 7 days post-I/R. **(O)** Interestingly, an upregulation of *Rho* was observed in the KO group compared to the WT group at 7 days post-I/R. Statistics were done with a two-way ANOVA followed by Tukey’s *post hoc* test. Data were presented as mean ± SEM in panels **(I,J,L)**. For RT-qPCR results, groups were compared using the pairwise fixed reallocation and randomization test. Data are shown as median ± quartile ± minimum/maximum in panels **(M–O)**. *p < 0.05, **p < 0.01; ***p < 0.001. *n* = 4–5/group. **(I,J)** The WT CO group was set to 100%. Scale bar = 20 μm. OS: outer segments; IS: inner segments; ONL: outer nuclear layer; OPL: outer plexiform layer; INL: inner nuclear layer.

Our scotopic ERG recordings revealed significantly reduced a-wave amplitudes after ischemia. Since these reduced responses derived from the rod-photoreceptors, this cell type should be investigated more closely. Hence, rhodopsin^+^ stained area was measured in control and ischemic WT and KO retinae at 3 (Figures 6A–D,I) and 7 days after I/R (Figures 6E–H,J). Our results indicated a comparable rhodopsin^+^ staining area at 3 days after ischemia in both genotypes compared to the corresponding control groups (WT CO vs. WT I/R, *p* = 0.49 and KO CO vs. KO I/R, *p* = 0.95; Figure 6I). Remarkably, the statistical evaluation of both ischemic groups verified a significantly diminished rhodopsin^+^ staining area in the WT I/R compared to the KO I/R group (*p* = 0.048). Also, an equal rhodopsin staining was found in both control groups at 7 days after I/R (*p* = 0.72; Figure 6J). However, the statistical analyses verified a significant lower rhodopsin immunoreactivity in ischemic WT retinae (WT CO vs. WT I/R, *p* = 0.04). In the KO condition no alterations were detectable at this point in time (KO CO vs. KO I/R, *p* = 0.98).

In addition, rhodopsin protein levels were determined via Western blot analyses at 7 days after ischemia. Therefore, we measured the band intensity of rhodopsin at ∼40 kDa (Figure 6K). We observed significantly reduced rhodopsin protein levels in WT I/R compared to both non-ischemic genotypes (WT I/R vs. WT CO, *p* = 0.012 and vs. KO CO, *p* = 0.008; Figure 6L). In a direct comparison of both ischemic groups, we noted a significant decrease of rhodopsin in the WT condition (*p* < 0.001). No differences were observed between the KO CO and the KO I/R groups (*p* = 0.32) as well as between the WT CO and KO CO groups (*p* = 0.94).

Finally, we analyzed the *rhodopsin* (*Rho*) expression via RT-qPCR at 3 and 7 days post-ischemia (Figures 6M–O). No changes in the *Rho* expression were found between non-ischemic WT and KO retinae at either point in time (3 days: WT CO vs. KO CO, *p* = 0.58; 7 days: WT CO vs. KO CO, *p* = 0.54; Figure 6M and Supplementary Table 2). A significant decrease of *Rho* mRNA levels was determined in WT I/R compared to WT CO at 3 days (WT CO vs. WT I/R, *p* = 0.04) as well as at 7 days after ischemia (WT CO vs. WT I/R, *p* = 0.01; Figure 6N). No significant alterations were seen between control and ischemic KO retinae (3 days: KO CO vs. KO I/R, *p* = 0.15; 7 days: KO CO vs. KO I/R, *p* = 0.14; Figure 6N). In a direct comparison of the two ischemic groups, a slightly enhanced *Rho* expression was found in the *Tnc* KO group at day 3 after ischemia (WT I/R vs. KO I/R, *p* = 0.33; Figure 5O). Interestingly, at 7 days, the *Rho* level was significantly enhanced in the ischemic KO condition (WT I/R vs. KO I/R, *p* = 0.049).

In order to further validate rod-photoreceptor degeneration, we analyzed the mRNA expression of another rod-photoreceptor marker, namely *rod transducin* [guanine nucleotide binding protein, alpha transducing 1 (*Gnat1*), Supplementary Table 2 and Supplementary Figures 4A–C]. Our analyses showed significantly reduced *Gnat1* expression levels in the WT I/R compared to the WT CO group at 7 days post-I/R (WT CO vs. WT I/R, *p* = 0.03; Supplementary Figure 4B). However, in line with the previous findings, no significant changes were observed in the KO CO compared to the KO I/R group or in the other groups (*p* = 0.66; Supplementary Figures 4A–C).

To follow the degeneration of photoreceptors more closely, apoptotic cells were labeled with an antibody directed against activated caspase 3 (Supplementary Figures 5A–E). In comparison to the CO groups, we found a significantly higher number of activated caspase 3^+^ cells in the ONL of both ischemic groups (WT CO vs. WT I/R, *p* < 0.001; KO CO vs. KO I/R, *p* < 0.001). However, a reduced number of apoptotic cells was noted in the ischemic KO compared to the ischemic WT group (WT I/R vs. KO I/R, *p* = 0.006).

In sum, our analyses revealed rod-photoreceptors damage after retinal ischemia. However, compared to the KO retina, a more pronounced damage was observed in the WT retina, indicating a better rod-photoreceptor outcome in the ischemic *Tnc* KO retina. These results suggest that the loss of Tnc not only partially preserves the function of rod-photoreceptors, but also improves their survival.

### Retinal Ischemia Impacts Glutamatergic Signaling

**FIGURE 7:**
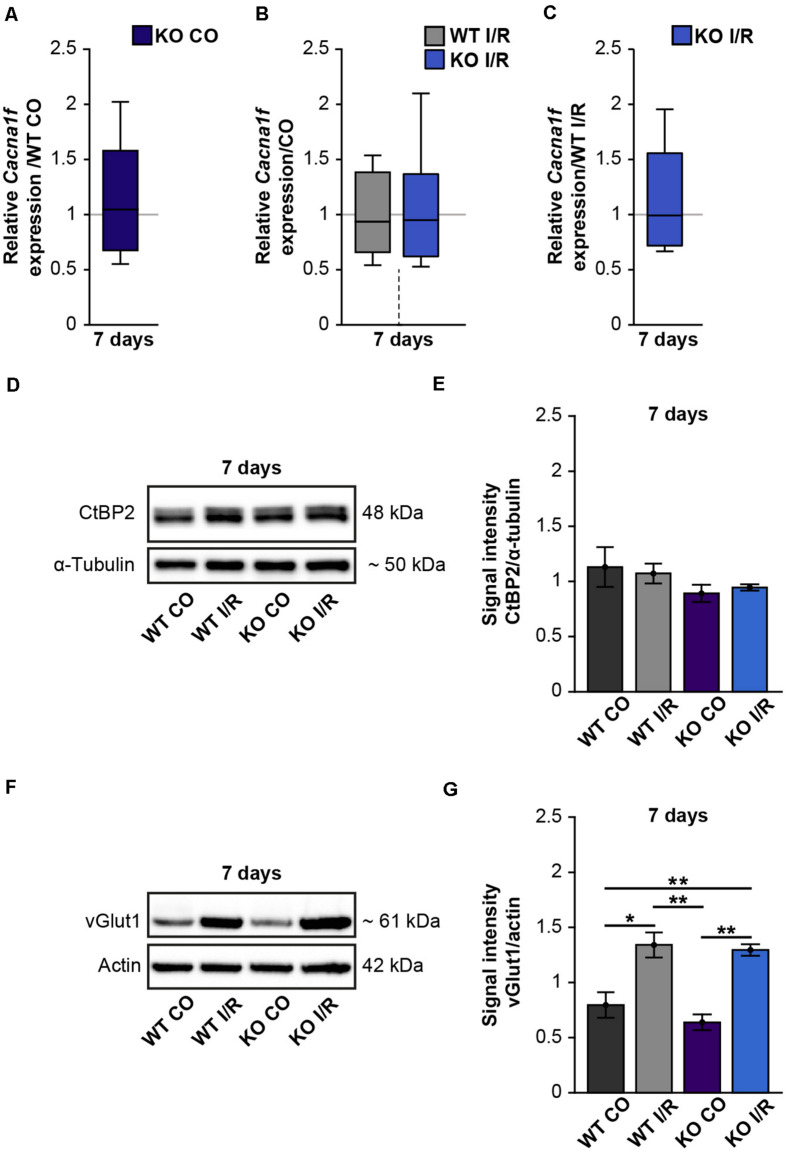
Enhanced protein levels of the pre-synaptic marker vGlut1 after retinal ischemia. **(A–C)** RT-qPCR analyses of the synaptic marker *Cacna1f* at 7 days after ischemia. **(A)** Equivalent mRNA expression levels were observed for *Cacnaf1* in the KO CO compared to the WT CO group. **(B)** Similar levels of *Cacnaf1* were measured in the WT I/R compared to the WT CO group as well as in the KO I/R compared to the KO CO group. **(C)** *Cacnaf1* mRNA expression levels were also found to be comparable in both ischemic groups. **(D)** Representative Western blots of CtBP2/ribeye at 7 days post-I/R. **(E)** Quantification of the band intensity revealed similar CtBP2 protein levels in the retina of all groups. **(F)** Exemplary Western blots of vGlut1 at 7 days post-I/R. **(G)** Quantification of the band intensity showed significantly elevated vGlut1 protein levels in the retina of both ischemic groups. For Western blot analyses, groups were analyzed using two-way ANOVA followed by Tukey’s *post hoc* test. These data are displayed as mean ± SEM in panels **(E,G)**. For RT-qPCR analyses, groups were analyzed using the pairwise fixed reallocation and randomization test and data in panels **(A–C)** are shown as median ± quartile ± minimum/maximum. *p < 0.05; **p < 0.01. *n* = 3–4/group.

Due to our findings of the impaired structural integrity of the OPL after ischemia and the OPL-associated expression of Tnc, we next focused on possible synaptic changes in the WT and KO retina. Previously, it was described that L-type voltage-gated calcium channels (LTCCs)-dependent synaptic plasticity is impaired in the hippocampus of Tnc deficient mice (Evers et al., 2002). Cav1.4 is a major LTCC at the pre-synaptic terminals of photoreceptors. It is crucial for the release of neurotransmitters (Strom et al., 1998) as well as for the formation of ribbon synapses, which constitute important contact points between rod-photoreceptors and bipolar cells (Liu X. et al., 2013). Additionally, Laird et al. (2019) noted that Cav1.4 expression is necessary for synaptic terminal development of rod-photoreceptors. Based on these reports and the initial results of our study, we hypothesized that ischemia and Tnc loss affect photoreceptor ribbon synapses. Therefore, we examined the mRNA expression of the *Cacna1f* gene, encoding the α1F calcium channel subunit of Cav1.4 (Morgans et al., 2005), in control and ischemic WT and KO retinae by RT-qPCR analyses (Figures 7A–C and Supplementary Table 2). Our investigations revealed no notable change in the *Cacna1f* expression level between both control groups (WT CO vs. KO CO, *p* = 0.84; Figure 7A). No alterations were observed between the ischemic and control groups (WT CO vs. WT I/R, *p* = 0.72; KO CO vs. KO I/R, *p* = 0.84; Figure 7B). Also, a comparable mRNA level of *Cacna1f* was found in both ischemic groups (WT I/R vs. KO I/R, *p* = 0.99). Additionally, we analyzed protein levels of the synaptic ribbon component ribeye [C-terminal binding protein 2 (CtBP2)] (Schmitz et al., 2000) by Western blot analyses (Figures 7D,E). CtBP2 levels were equivalent in both control groups (WT CO vs. KO CO, *p* = 0.99) as well as in both ischemic groups (WT I/R vs. KO I/R, *p* = 0.99; Figure 7E). Also, the comparison of the control and ischemic groups (WT CO vs. WT I/R, *p* = 0.86; KO CO vs. KO I/R, *p* = 0.60; Figure 7E) revealed very similar CtBP2 protein levels.

Photoreceptors and bipolar cells transfer visual information via the release of excitatory glutamate at ribbon synapses (Brandstatter and Hack, 2001; tom Dieck and Brandstatter, 2006). Previous studies could demonstrate that the vGlut1 is associated with pre-synaptic terminals of photoreceptor and bipolar cells and required for their signaling (Johnson et al., 2003; Sherry et al., 2003; Johnson J. et al., 2007). Interestingly, in comparison to the respected control groups, we noted significantly increased vGlut1 protein levels in both ischemic groups (WT CO vs. WT I/R, *p* = 0.01; KO CO vs. KO I/R, *p* = 0.001; Figures 7F,G). Although vGlut1 protein levels were comparable in the WT I/R and KO I/R group (WT I/R vs. KO I/R, *p* = 0.98).

In sum, these results suggest that glutamatergic signaling is impaired after retinal ischemia but not different in the retina of WT and KO mice.

## Discussion

In the present study, we used a KO mouse I/R model to analyze the functional importance of the ECM component Tnc in retinal neurodegeneration. Previously, Tnc dysregulation has been described not only after cerebral, but also hepatic and myocardial ischemic degeneration (Lu et al., 2003; Taki et al., 2010, 2015; Kuriyama et al., 2011). However, the role of Tnc in retinal ischemia is not yet understood.

Regarding the expression pattern, we found that extracellular Tnc associates with horizontal and amacrine cells and observed a synaptic layer-associated immunoreactivity in the OPL and IPL, even after ischemia. Additionally, we identified Tnc and GFAP co-expressing astrocytes in the ischemic retina, indicating that also reactive astrocytes are a cellular source of this ECM component. These observations are consistent with previous reports on the Tnc expression pattern in the healthy chick and mouse retina (Bartsch et al., 1995; D’Alessandri et al., 1995; Reinhard et al., 2015).

We noted an increased Tnc immunoreactivity 3 days after I/R, but interestingly there was a significant reduction after 7 days. Via Western blot analyses, we were able to determine a specific increase of larger Tnc isoforms (>250 kDa). However, we could not detect any significant differences regarding Tnc protein levels 7 days post-I/R, indicating that protein levels drop back to a baseline level shortly after injury. Enhanced Tnc levels were reported in subarachnoid hemorrhage (Shiba et al., 2014; Suzuki et al., 2020), ischemic brain injury (Manrique-Castano et al., 2020) and acute cortical laser lesion (Roll et al., 2012). In the lesioned brain, an increase of relative *Tnc* expression could only be detected for a short time, while enhanced protein levels were found 3 and 7 days post-lesion, suggesting that high Tnc protein levels exceeded the brief upregulation seen on mRNA level (Roll et al., 2020). Furthermore, a temporally Tnc regulation was observed under retinal degenerative conditions. A significant increase of Tnc immunoreactivity was found in an experimental autoimmune glaucoma model at 7 and 14 days, whereas no differences were observed at a later point in time (Reinehr et al., 2016). In an IOP-dependent glaucoma model, however, we recently observed increased Tnc levels a few weeks after IOP elevation (Reinhard et al., 2021), which suggests that Tnc expression depends not only on the time, but also on the type of retinal damage. In an I/R rat model, we detected diminished levels of Tnc at 21 days post-ischemia. In this model, the loss of Tnc expressing amacrine and horizontal cells as a result of progressive ischemic damage could explain the Tnc dysregulation (Reinhard et al., 2017a). This might also correspond to our findings of a decreased Tnc immunoreactivity at 7 days after ischemia.

Based on our findings regarding equal mRNA and time-dependent altered protein levels, we propose that Tnc dysregulation under ischemic conditions takes place on translational or post-translation level. Our Western blot analyses indicate increased levels of larger Tnc isoforms after ischemia. *Vice versa* shorter isoforms were found unaltered. So far, 28 Tnc isoforms have been identified in the CNS (Joester and Faissner, 1999; von Holst et al., 2007). Previous findings revealed that the shorter Tnc variants are preferentially expressed in the healthy CNS, while larger forms dominate after cortical lesion. Here, a strong upregulation of the alternative spliced fibronectin III domains B and D was described 2–4 days post-lesion (Dobbertin et al., 2010). In a future perspective, it would be therefore interesting to perform comprehensive mRNA as well as protein analyses on specific Tnc isoforms, in particular after ischemic damage.

The main focus of our study was to comparatively explore retinal function and rod-bipolar/photoreceptor cell degeneration in WT and *Tnc* KO mice after ischemic insult. Previous studies already described that the functional restriction increased over time in ischemia rat models with an ischemia duration between 30 and 90 min (Jehle et al., 2008; Schmid et al., 2014; Palmhof et al., 2019). However, Zhao et al. (2013) could show that a moderate ischemia of only 17 min does not affect photoreceptors. To investigate functional deficits in the retina of our model, we performed ERG recordings 7 days post-ischemia. Here, mice underwent 45 min of I/R. Under scotopic conditions, a-wave amplitudes were significantly reduced after ischemia, indicating a diminished rod-photoreceptor response in both genotypes. Although not statistically significant, we observed a trend toward increased a-wave amplitudes in the ischemic KO compared to the WT group. This protective effect was even more pronounced in regard to the b-wave amplitudes, which display the response of rod-bipolar cells. In accordance with our results, it is well documented that the b-wave in particular is very sensitive to ischemic retinal injury (Osborne et al., 2004). We observed a significant b-wave amplitude decrease for all light flash luminances in the ischemic WT group compared to both non-ischemic control groups. Remarkably, a better outcome of the b-wave responses was observed after I/R in KO mice compared to the control groups. The ischemia-induced decrease was statistically significant in the WT compared to the KO. Overall, our analyses revealed that retinal function was clearly more affected in the presence of Tnc. Therefore, we suggest that the absence of Tnc may have a protective effect on rod-photoreceptors and rod-bipolar cells after ischemic damage in the retina. Reduced a-wave amplitudes indicate an impairment of rod-mediated photoreceptor function post-I/R.

In our study, we found unaltered recoverin levels, but a time-dependent decrease of rhodopsin mRNA and protein levels in the WT I/R group. As previously described, KO of rhodopsin abolished rod-driven electrophysical activity (Xiao et al., 2019). Moreover, a connection between altered rhodopsin levels and neurodegenerative processes as well as the use of rhodopsin as a potential early biomarker have been reviewed previously by Lenahan et al. (2020). In contrast, although *Rho* mRNA levels were also observed significantly lower, an unaltered rhodopsin immunoreactivity was found in ischemic rat models (Zhao et al., 2013; Palmhof et al., 2019). This indicates that the degeneration of rod-photoreceptor cells severely depends on the experimental conditions. However, our results demonstrate that the loss of Tnc prevents rhodopsin/*Rho* as well as *Gnat1* downregulation in rod-photoreceptor cells. This result is also reflected by the better a-wave amplitude outcome and a lower number of apoptotic cells in the ONL of ischemic *Tnc* KO mice. Our ERG recordings also revealed a significant reduction of b-wave amplitudes, indicating that rod-bipolar cells could also be affected after ischemia. However, we observed no change in the number of PKCα^+^ rod-bipolar cells. This is in line with results of a previous study, which revealed no rod-bipolar cell loss after retinal ischemia in rats (Palmhof et al., 2019). Based on these overall findings, we propose that rod-bipolar cells are more robust against retinal ischemia on a structural level than rod-photoreceptors, but not on a functional level.

We also investigated the retinal structure by measuring layer thickness. Here, we found that the OPL was particularly diminished even 3 days post-I/R. It is well known that ischemia-induced damage is associated with a disorganization as well as time-dependent thinning of retinal layers (Palmhof et al., 2019). We observed retinal thinning in WT I/R mice after 3 and 7 days. Although compared to the KO CO group, retinal thinning was not observed in the KO I/R group until 7 days. Therefore, our H&E stainings point to a time-delayed damage of the retinal layers in *Tnc* KO mice after I/R. Only a moderate thinning of the ONL was observed at 7 days after I/R. However, based on the increased number of apoptotic cells that we observed in the ONL, we expect a more progressive thinning at later points in time. As stated, we noted a thinning of the OPL. Thus, we speculate that this thinning could be caused by synaptic remodeling, which might precede the ONL thinning.

Tenascin-C has been reported to be implicated in synapse development and synaptic plasticity (Evers et al., 2002; Heikkinen et al., 2014; Gottschling et al., 2019). Based on these reports and due to our finding of reduced a- and b-wave amplitudes and severe reduction of the OPL after ischemia, we also examined the signal transfer from photoreceptors to bipolar cells by analyzing the expression of various synaptic proteins. Our analyses of *Cacna1f* and CtBP2 revealed comparable mRNA/protein levels in the WT and KO control and ischemic groups. Thus, these findings suggest that neither ischemia nor the loss of Tnc has a huge influence on photoreceptor/bipolar cell ribbon synapses. However, in this regard, immunohistochemical as well as ultrastructural analyses of ribbon synapses after ischemia and Tnc loss are necessary to explore a possible impact.

In our study, we observed a significant upregulation of vGlut1 protein levels in the ischemic WT and KO retinae. Since vGlut1 is predominantly expressed in the pre-synaptic terminals of photoreceptor and bipolar cells and important for signaling transmission (Johnson et al., 2003; Sherry et al., 2003; Johnson J. et al., 2007), elevated vGlut1 levels after ischemia strongly suggest an impaired glutamatergic photoreceptor-bipolar cell signaling. In retinal ischemia, abnormalities in the glutamate metabolism, including a dysregulation of various glutamate transporters, have been previously described (Bringmann et al., 2013; Ishikawa, 2013). However, *vGlut1* expression level alterations were not reported in a mouse model of central retinal artery occlusion and retinal ischemia 1 day after damage induction (Michalski et al., 2013). This discrepancy could again probably be explained by the examined time points as well as experimental conditions of the ischemic insult. VGlut1 plays an important role in packaging excitatory glutamate for synaptic release. Therefore, elevated vGlut1 levels could also be associated with glutamate excitotoxicity and apoptosis, which we also observed in our I/R model. Increased vGlut1 protein levels may also play a neuroprotective role, as a controlled excitatory glutamate storage is crucial to avoid neurotoxicity and to provide a normal signaling transfer from e.g., photoreceptors to bipolar cells (Yang, 2004; Omote et al., 2011; Liu K. et al., 2013). Additionally, elevated vGlut1 levels may point to plasticity by means of neuronal circuits reconstruction after ischemic insult (Kim et al., 2005). Previously, analyses of *vGlut1* KO mice could show that vGlut1 is required for synaptic signaling from photoreceptors to retinal output neurons but not for intrinsic visual functions (Johnson J. et al., 2007). ERG recordings in *vGlut1* KO mice revealed that no photoreceptor-driven visual signals are transmitted to ON bipolar cells. Thus, an overall imbalance of vGlut1 levels could also explain the impaired a- and b-wave amplitudes that we observed after ischemia. Importantly, we did not find any significant differences between ischemic WT and KO mice, which might indicate that the increase of vGlut1 is rather related to the ischemic damage than the Tnc deficiency.

## Conclusion

Our results revealed an early, but short-term, upregulation of Tnc in the ischemic retina. Most importantly, our data indicate that ischemic *Tnc* KO mice have a better outcome in regard to retinal functionality and integrity as well as survival of rod-photoreceptor cells. Furthermore, increased vGlut1 levels after ischemia could point to an impaired glutamatergic photoreceptor-bipolar cell signaling and excitotoxicity. In summary, our findings suggest that the induction of the ECM glycoprotein Tnc contributes to ischemia-induced degenerative processes in the retina, possibly also by remodeling of synaptic sites. A better understanding of ECM remodeling and synaptic changes associated with retinal ischemia could be useful for the development of novel neuroprotective strategies.

## Data Availability Statement

The original contributions presented in the study are included in the article/Supplementary Material, further inquiries can be directed to the corresponding author/s.

## Ethics Statement

Animal experiments adhered to the “Association for Research and Vision and Ophthalmology (ARVO)” Statement for the Use of Animals in Ophthalmic and Vision Research. All animal care and experimental procedures were conducted in accordance with the EU animal welfare protection laws and regulations and were approved by the Ethics Committee of the state North Rhine-Westphalia, Germany (AZ 81-02.04.2018.A194). The study was supervised by the animal welfare commissioner of the Ruhr-University Bochum.

## Author Contributions

SW and JR wrote the manuscript. SW, AY, CP, AM-B, NW, and JR performed the experiments. SW, AY, CP, AM-B, and JR analyzed the data. SJ revised the manuscript. JR designed the study. All authors read and approved the final manuscript.

## Conflict of Interest

The authors declare that the research was conducted in the absence of any commercial or financial relationships that could be construed as a potential conflict of interest.

## Abbreviations

BSA: bovine serum albumin
CNS: central nervous system
CtBP2: C-terminal binding protein 2
ECM: extracellular matrix
ERG: electroretinogram
GCL: ganglion cell layer
*Gnat1*: *guanine nucleotide binding protein, alpha transducin 1*
H&E: Hematoxylin and Eosin
*Hprt*: *hypoxanthine guanine phosphoribosyl transferase*
INL: inner nuclear layer
IOP: intraocular pressure
IPL: inner plexiform layer
IS: inner segment
KO: knock-out
KO CO: non-ischemic control *Tnc* knock-out group
KO I/R: ischemic *Tnc* knock-out group
LTCC: L-type voltage-gated calcium channel
NFL: nerve fiber layer
ONL: outer nuclear layer
OPL: outer plexiform layer
OS: outer segment
PBS: phosphate-buffered saline
PFA: paraformaldehyde
PKC α /*Prkca*: protein kinase alpha C
RT: room temperature
RT-qPCR: quantitative real time PCR
SEM: standard error mean
TBS: Tris-buffered saline
Tnc: tenascin-C
vGlut1: vesicular glutamate transporter 1
WT: wild type
WT CO: non-ischemic control wild type group
WT I/R: ischemic wild type group.
