# Supplementary figures and images for: Knock-Out of Tenascin-C Ameliorates Ischemia-Induced Rod-Photoreceptor Degeneration and Retinal Dysfunction
Source: Front Neurosci. 2021 May 20;15:642176. doi: 10.3389/fnins.2021.642176 (PMC8172977; doi:10.3389/fnins.2021.642176)

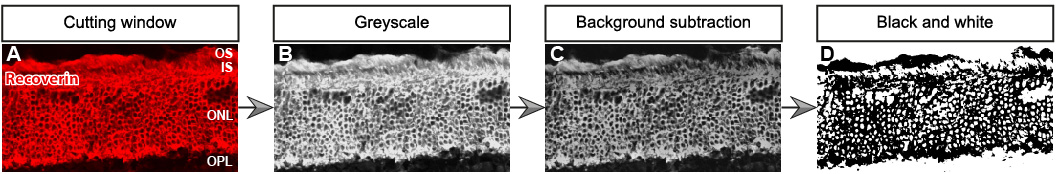

Supplement: Supplementary file 1 [file Image_1.JPEG]

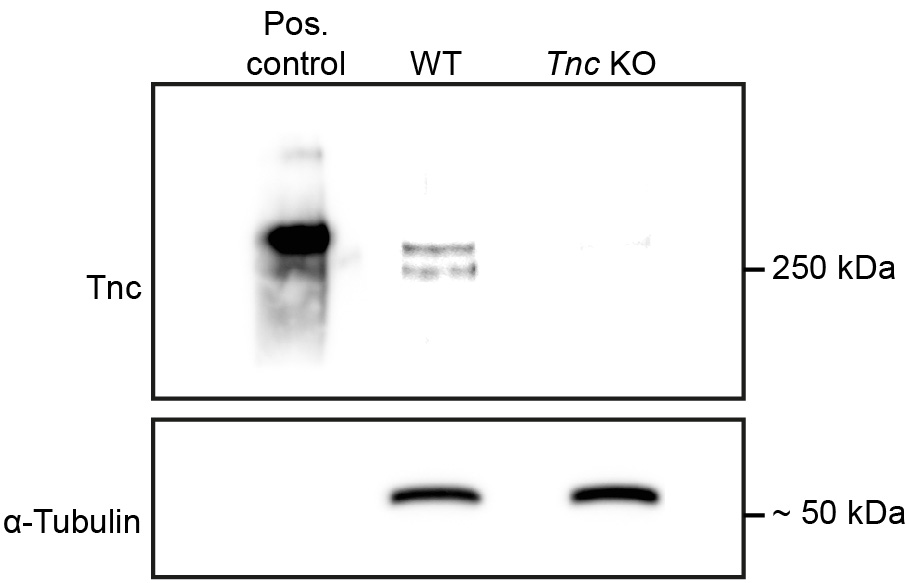

Supplement: Supplementary file 2 [file Image_2.JPEG]

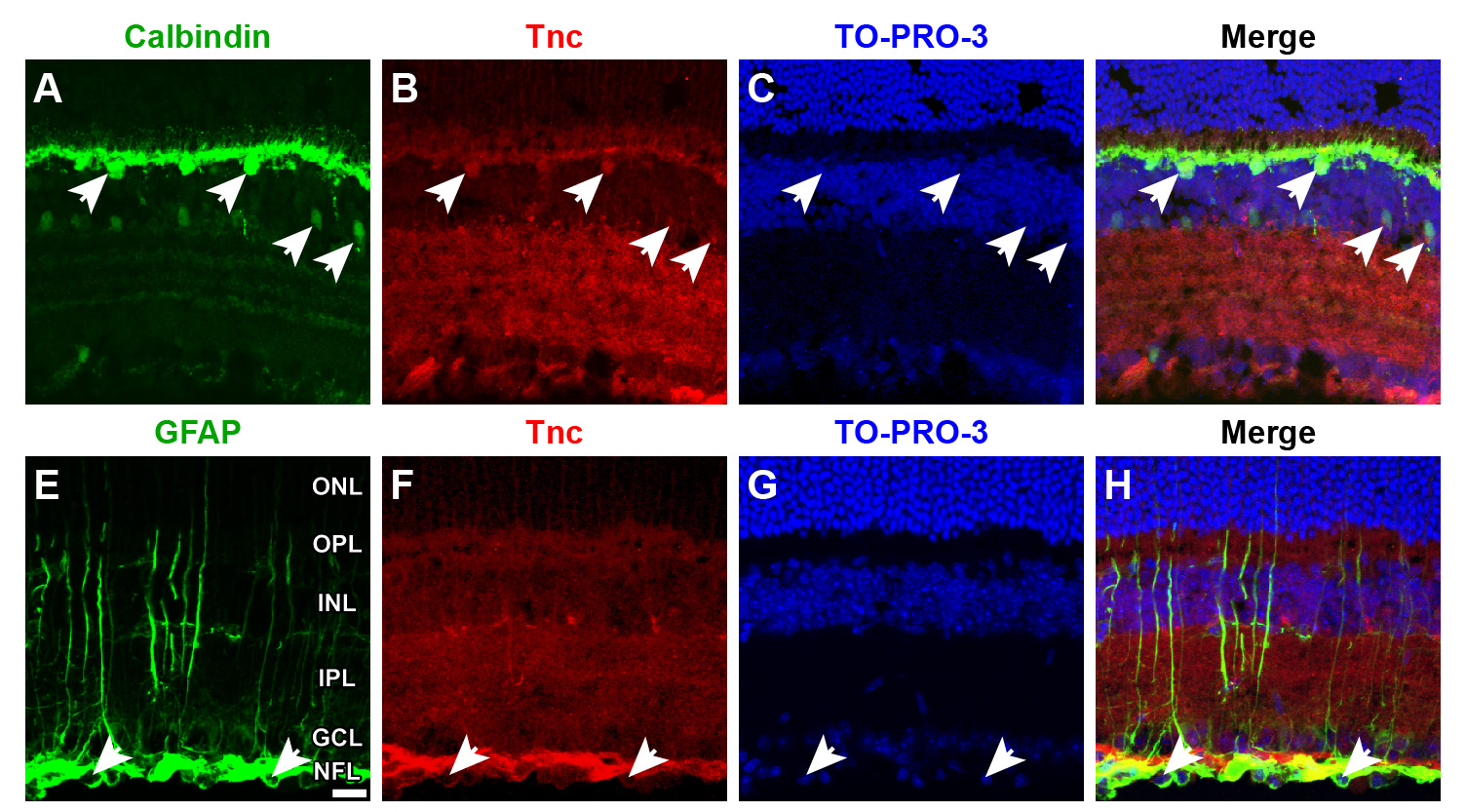

Supplement: Supplementary file 3 [file Image_3.JPEG]

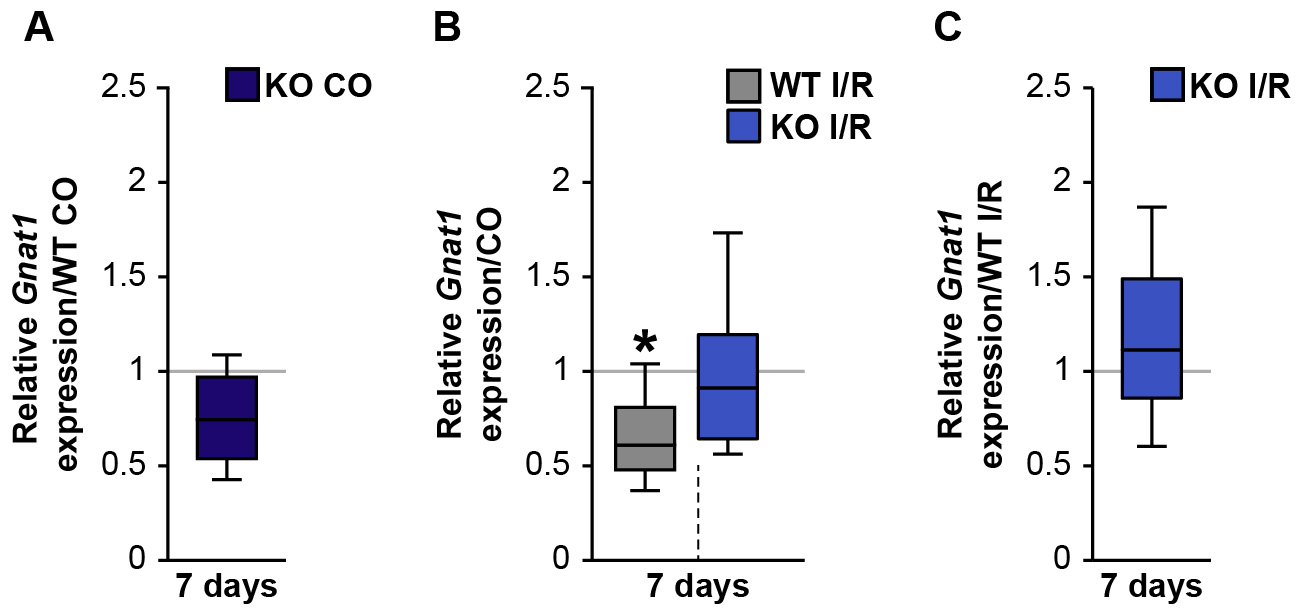

Supplement: Supplementary file 4 [file Image_4.JPEG]

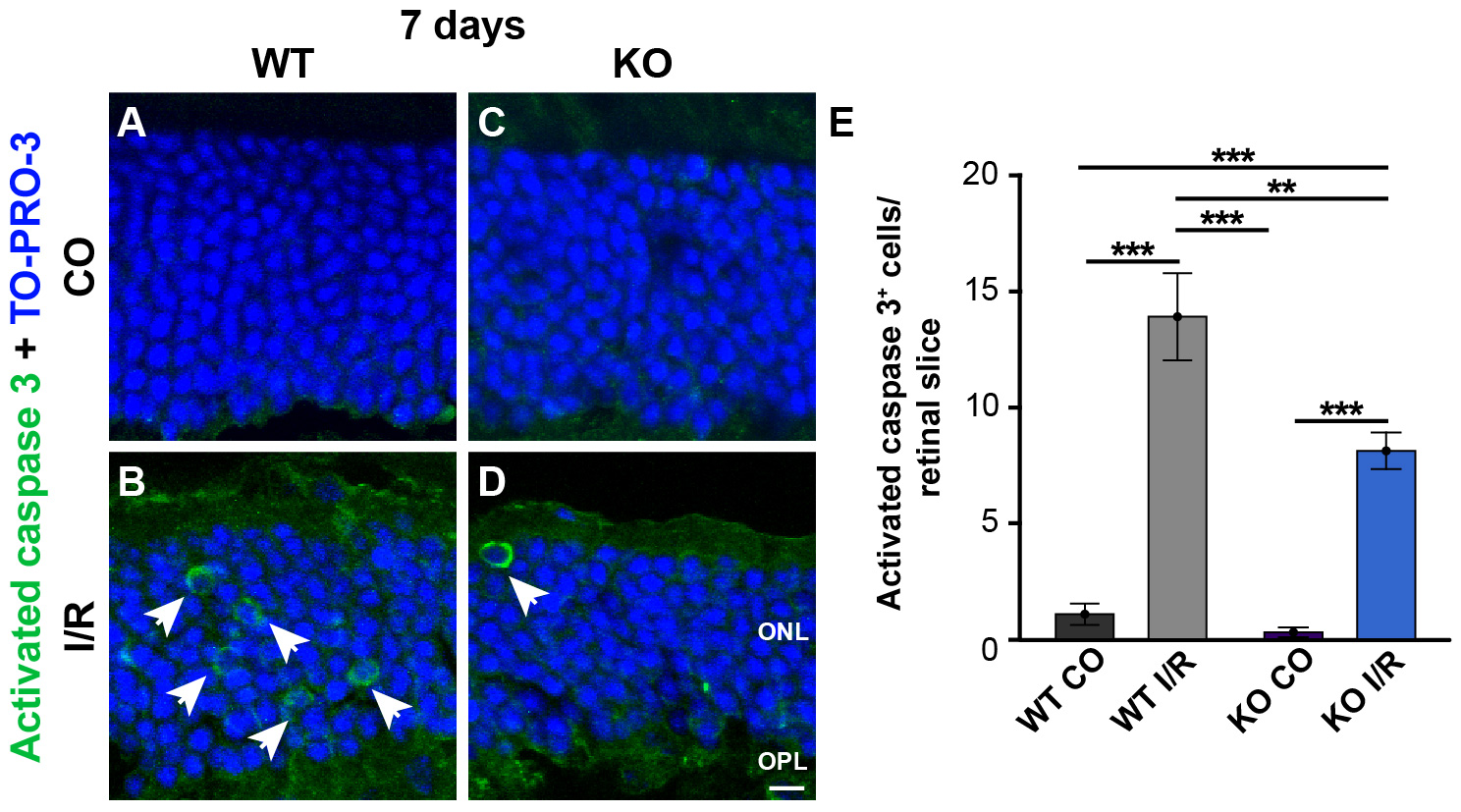

Supplement: Supplementary file 5 [file Image_5.JPEG]
