# Supplementary material for: Knock-Out of Tenascin-C Ameliorates Ischemia-Induced Rod-Photoreceptor Degeneration and Retinal Dysfunction
Source: Front Neurosci. 2021 May 20;15:642176. doi: 10.3389/fnins.2021.642176 (PMC8172977; doi:10.3389/fnins.2021.642176)
Supplement: Supplementary file 6 [file Data_Sheet_1.docx]

Supplement Material

# Supplement Tables

**Supplement Table 1:** Adjustments of the ImageJ macro to analyze the immunopositive area [%].

| **Protein** | **Cutting window/image** | **Background**  **subtraction** | **Lower**  **threshold** | **Upper**  **threshold** |
| --- | --- | --- | --- | --- |
| Recoverin | 220 µm x 120 µm | 50 | 23.68 | 78.72 |
| Rhodopsin | 220 µm x 66 µm | 50 | 27.82 | 97.28 |
| Tnc | 185 µm x 185 µm | 50 | 12.49 | 76.91 |

**Supplement Table 2:** Expression analyses in control and ischemic retinae of WT and *Tnc* KO mice 3 and 7 days post I/R by RT-qPCR analyses. P-values < 0.05 are shown in bold.

| **Genotype/**  **Group** | | **Gene** | **Median**  **(fold-change)** | | **Quartile ± minimum/maximum** | | **P-value** | | **N** | |
| --- | --- | --- | --- | --- | --- | --- | --- | --- | --- | --- |
| **3 days** | | | | | | | | | | |
| WT CO vs. WT I/R | | *Prkca* | 0.737 | | 0.458 – 1 025 | | 0.183 | | 5 | |
|  |  |  |  |  | 0.387 – 1.341 | |  |  |  |  |
| WT CO vs. KO CO | |  | 0.964 | | 0.720 – 1.228 | | 0.783 | |  |  |
|  |  |  |  |  | 0.664 – 1.484 | |  |  |  |  |
| KO CO vs. KO I/R | |  | 0.751 | | 0.464 – 1.180 | | 0.199 | |  |  |
|  |  |  |  |  | 0.347 – 1.372 | |  |  |  |  |
| WT I/R vs. KO I/R | |  | 0.983 | | 0.535 – 1.779 | | 0.906 | |  |  |
|  |  |  |  |  | 0.383 – 2.354 | |  |  |  |  |
| WT CO vs. WT I/R | | *Rcvrn* | 0.701 | | 0.532 – 1.044 | | 0.072 | | 5 | |
|  |  |  |  |  | 0.386 – 1.179 | |  |  |  |  |
| WT CO vs. KO CO | |  | 0.909 | | 0.620 – 1.353 | | 0.641 | |  |  |
|  |  |  |  |  | 0.417 – 1.726 | |  |  |  |  |
| KO CO vs. KO I/R | |  | 0.908 | | 0.626 -1.313 | | 0.616 | |  |  |
|  |  |  |  |  | 0.506 – 2.090 | |  |  |  |  |
| WT I/R vs. KO I/R | |  | 1.176 | | 0.805 – 1.618 | | 0.319 | |  |  |
|  |  |  |  |  | 0.790 – 2.256 | |  |  |  |  |
| WT CO vs. WT I/R | | *Rho* | 0.55 | | 0.349 – 0.935 | | **0.040** | | 5 | |
|  |  |  |  |  | 0.243 – 1.205 | |  |  |  |  |
| WT CO vs. KO CO | |  | 1.109 | | 0.794 – 1.562 | | 0.575 | |  |  |
|  |  |  |  |  | 0.546 – 2.026 | |  |  |  |  |
| KO CO vs. KO I/R | |  | 0.678 | | 0.393 – 1.134 | | 0.149 | |  |  |
|  |  |  |  |  | 0.305 – 1.664 | |  |  |  |  |
| WT I/R vs. KO I/R | |  | 1.364 | | 0.769 – 2.414 | | 0.325 | |  |  |
|  |  |  |  |  | 0.502 – 3.978 | |  |  |  |  |
| WT CO vs. WT I/R | | *Tnc* | 0.973 | | 0.816 – 1.185 | | 0.782 | | 4 | |
|  |  |  |  |  | 0.668 – 1.334 | |  |  |  |  |
| **7 days** | | | | | | | | | | |
| WT CO vs. WT I/R | *Cacna1f* | | | 0.937 | | 0.658 – 1.385 | | 0.716 | | 4 |
|  |  |  |  |  |  | 0.542 – 1.538 | |  |  |  |
| WT CO vs. KO CO |  |  |  | 1.047 | | 0.677 – 1.580 | | 0.844 | |  |
|  |  |  |  |  |  | 0.551 – 2.023 | |  |  |  |
| KO CO vs. KO I/R |  |  |  | 0.951 | | 0.620 – 1.368 | | 0.835 | |  |
|  |  |  |  |  |  | 0.528 – 2.099 | |  |  |  |
| WT I/R vs. KO I/R |  |  |  | 0.993 | | 0.719 – 1.558 | | 0.985 | |  |
|  |  |  |  |  |  | 0.667 – 1.956 | |  |  |  |
| WT CO vs. WT I/R | *Gnat1* | | | 0.611 | | 0.479 – 0.810 | | 0.025 | | 4 |
|  |  |  |  |  |  | 0.396 – 1.039 | |  |  |  |
| WT CO vs. KO CO |  |  |  | 0.745 | | 0.539 – 0.970 | | 0.098 | |  |
|  |  |  |  |  |  | 0.427 – 1.088 | |  |  |  |
| KO CO vs. KO I/R |  |  |  | 0.913 | | 0.643 – 1.194 | | 0.664 | |  |
|  |  |  |  |  |  | 0.563 – 1.733 | |  |  |  |
| WT I/R vs. KO I/R |  |  |  | 1.114 | | 0.859 – 1.491 | | 0.587 | |  |
|  |  |  |  |  |  | 0.604 – 1.871 | |  |  |  |
|  |  |  |  |  |  | 0.515 – 1.183 | |  |  |  |
| WT CO vs. WT I/R | *Prkca* | | | 0.807 | | 0.503 – 1.396 | | 0.351 | | 5 |
|  |  |  |  |  |  | 0.343 – 1.662 | |  |  |  |
| WT CO vs. KO CO |  |  |  | 0.757 | | 0.484 – 1.246 | | 0.317 | |  |
|  |  |  |  |  |  | 0.288 – 1.867 | |  |  |  |
| KO CO vs. KO I/R |  |  |  | 0.962 | | 0.601 – 1.637 | | 0.862 | |  |
|  |  |  |  |  |  | 0.454 – 2.335 | |  |  |  |
| WT I/R vs. KO I/R |  |  |  | 0.902 | | 0.590 -1.317 | | 0.591 | |  |
|  |  |  |  |  |  | 0.528 – 1.894 | |  |  |  |
| WT CO vs. WT I/R | *Rcvrn* | | | 0.924 | | 0.694 – 1.212 | | 0.516 | | 5 |
|  |  |  |  |  |  | 0.619 – 1.263 | |  |  |  |
| WT CO vs. KO CO |  |  |  | 0.923 | | 0.645 – 1.336 | | 0.673 | |  |
|  |  |  |  |  |  | 0.513 – 1.744 | |  |  |  |
| KO CO vs. KO I/R |  |  |  | 0.971 | | 0.678 – 1.337 | | 0.844 | |  |
|  |  |  |  |  |  | 0.468 – 1.853 | |  |  |  |
| WT I/R vs. KO I/R |  |  |  | 0.969 | | 0.672 – 1.404 | | 0.569 | |  |
|  |  |  |  |  |  | 0.646 – 1.462 | |  |  |  |
| WT CO vs. WT I/R | *Rho* | | | 0.511 | | 0.313 – 0.752 | | **0.012** | | 5 |
|  |  |  |  |  |  | 0.274 – 1.050 | |  |  |  |
| WT CO vs. KO CO |  |  |  | 1.119 | | 0.764 – 1.553 | | 0.542 | |  |
|  |  |  |  |  |  | 0.674 – 2.131 | |  |  |  |
| KO CO vs. KO I/R |  |  |  | 0.740 | | 0.514 – 1.115 | | 0.140 | |  |
|  |  |  |  |  |  | 0.378 – 1.281 | |  |  |  |
| WT I/R vs. KO I/R |  |  |  | 1.695 | | 1.064 – 2.987 | | **0.049** | |  |
|  |  |  |  |  |  | 0.782 – 3.475 | |  |  |  |
| WT CO vs. WT I/R | *Tnc* | | | 0.899 | | 0.689 – 1.238 | | 0.581 | | 4 |
|  |  |  |  |  |  | 0.514 – 1.519 | |  |  |  |

| **Flash luminances**  **[cd*s/m²]** | **1** | | **3** | | **10** | | **25** | |
| --- | --- | --- | --- | --- | --- | --- | --- | --- |
| **Amplitude [µV]** | **Mean** | **SEM** | **Mean** | **SEM** | **Mean** | **SEM** | **Mean** | **SEM** |
| WT CO (1) | 173.80 | 18.84 | 210.58 | 16.58 | 268.46 | 13.96 | 270.26 | 13.09 |
| WT I/R (2) | 38.30 | 14.18 | 56.34 | 21.21 | 92.10 | 69.48 | 97.04 | 57.85 |
| KO CO (3) | 142.46 | 18.95 | 181.04 | 16.38 | 225.44 | 32.04 | 231.04 | 24.33 |
| KO I/R (4) | 59.98 | 16.21 | 89.90 | 21.88 | 131.80 | 23.70 | 150.58 | 14.19 |
| P-value 1 vs. 2 | **0.001** | | **0.001** | | **0.03** | | **0.009** | |
| P-value 1 vs. 3 | 0.58 | | 0.70 | | 0.88 | | 0.83 | |
| P-value 1 vs. 4 | **0.001** | | **0.002** | | 0.12 | | 0.09 | |
| P-value 2 vs. 3 | **0.003** | | **0.002** | | 0.14 | | **0.047** | |
| P-value 2 vs. 4 | 0.81 | | 0.61 | | 0.90 | | 0.66 | |
| P-value 3 vs. 4 | **0.02** | | **0.02** | | 0.39 | | 0.34 | |
| **Amplitude [µV]** | **Mean** | **SEM** | **Mean** | **SEM** | **Mean** | **SEM** | **Mean** | **SEM** |
| WT CO (1) | 504.90 | 46.55 | 515.42 | 36.48 | 577.70 | 34.51 | 612.18 | 28.48 |
| WT I/R (2) | 103.42 | 23.22 | 73.26 | 14.53 | 68.06 | 18.16 | 92.54 | 24.42 |
| KO CO (3) | 513.70 | 80.76 | 539.58 | 79.61 | 560.88 | 96.05 | 599.38 | 107.58 |
| KO I/R (4) | 287.20 | 55.55 | 357.74 | 75.47 | 381.16 | 78.85 | 395.58 | 71.63 |
| P-value 1 vs. 2 | **0.001** | | **0.001** | | **0.001** | | **0.001** | |
| P-value 1 vs. 3 | 1.00 | | 0.99 | | 1.00 | | 1.00 | |
| P-value 1 vs. 4 | 0.06 | | 0.26 | | 0.18 | | 0.15 | |
| P-value 2 vs. 3 | **0.001** | | **0.001** | | **0.001** | | **0.001** | |
| P-value 2 vs. 4 | 0.13 | | **0.02** | | **0.02** | | **0.03** | |
| P-value 3 vs. 4 | **0.048** | | 0.16 | | 0.25 | | 0.18 | |

**Supplement Table 3:** Scotopic electroretinogram recordings from control and ischemic WT and *Tnc* KO retinae 7 days after I/R. P-values < 0.05 are shown in bold.

**Supplement Figure 1:** Image processing using ImageJ software. An exemplary recoverin stained WT CO retina image is shown. **(A)** First, cropped image files (Table 2) were imported to the ImageJ software. **(B)** The images were then converted into 32-bit greyscale. **(C)** Next, a previously defined appropriate background was subtracted. **(D)** Finally, the images were converted into black and white and the upper and lower thresholds (Table 2) were set. Appropriate thresholds were defined when the black and white picture matched the original image. The percentage of the area fraction was measured. OS: outer segments; IS: inner segments; ONL: outer nuclear layer; OPL: outer plexiform layer.

**Supplement Figure 2:** Specificity of the Tnc antibody. Specificity of the polyclonal Tnc antibody (KAF12; Wiemann et al., 2020) was shown by Western blot analyses. Immunoaffinity-purified mouse Tnc protein (0.25 µg/lane) from postnatal mouse brains was used as a positive control (pos. control). The Tnc antibody detects two specific bands at ~ 250 and > 250 kDa in the immunoaffinity-purified Tnc protein lysate. Additionally, we used retinal protein of WT and *Tnc* KO mice. The Tnc antibody also detects the two bands in the WT condition, while no Tnc protein was observed in the KO condition. Equal protein loading of retinal lysates was verified by the detection of α-tubulin (~ 50 kDa). No α-tubulin band was observed in the immunoaffinity-purified Tnc protein fraction, demonstrating the purity of the Tnc protein purification.

**Supplement Figure 3**: Horizontal, amacrine cells and astrocytes are a source of Tnc in the retina. **(A-D)** Coimmunostaining of calbindin^+^ cells (green) and Tnc (red) in the WT retina at 3 days after ischemia. We detected double positive horizontal cells. Additionally, we found Tnc immunoreactivity closely related to amacrine cells. **(E-F)** Colocalization of GFAP^+^ astrocytes (green) and Tnc (red) in the WT I/R retina after 3 days. **(C, G)** TO-PRO-3 (blue) was used as nuclear counterstain. White arrows indicate exemplary coimmunostaining. Scale bar = 20 µm. ONL: outer nuclear layer; OPL: outer plexiform layer; INL: inner nuclear layer; IPL: inner plexiform layer; GCL: ganglion cell layer; NFL: nerve fiber layer.

**Supplement Figure 4:** Retained *Gnat1* expression in the KO I/R retina. **(A-C)** RT-qPCR analyses of the rod-photoreceptor marker *Gnat1* (*rod transducin)* at 7 days after retinal ischemia. **(A)** No different *Gnat1* expression was noted in the KO CO compared to the WT CO group. **(B)** However, our analyses revealed significantly reduced levels of *Gnat1* mRNA levels in the WT I/R compared to the WT CO group, while a comparable expression was found in the KO I/R compared to the KO CO group. **(C)** A comparable *Gnat1* expression was observed in both ischemic groups. For statistical analyses of RT-qPCR data, groups were compared with a pairwise fixed reallocation and randomization test. Data are shown as median ± quartile ± minimum/maximum. *p < 0.05. n = 4/group.

**Supplement Figure 5:** Reduced apoptosis in the KO I/R retina. **(A-D)** Apoptotic cells were labeled with activated caspase 3 (green) in control and ischemic WT and KO retinae at 7 days after I/R. TO-PRO-3 (blue) was used as nuclear counterstain. **(E)** Compared to the CO groups, a significantly increased number of activated caspase 3^+^ cells was noted in the ONL of both ischemic groups. Although a reduced number of apoptotic cells was observed in the WT I/R in comparison to the KO I/R group. Analyzes were performed with a two-way ANOVA followed by Tukey`s post hoc test. Data were presented as mean ± SEM. **p < 0.01, ***p < 0.001. n = 4-6/group. Scale bar = 20 µm. ONL: outer nuclear layer; OPL: outer plexiform layer.
